# Proof Complexity of Modal Resolution

**DOI:** 10.1007/s10817-021-09609-9

**Published:** 2021-10-13

**Authors:** Sarah Sigley, Olaf Beyersdorff

**Affiliations:** 1 School of Computing, University of Leeds, Leeds, UK; 2 Institute of Computer Science, Friedrich Schiller University Jena, Jena, Germany

**Keywords:** Modal logic, Resolution, Proof complexity, Lower bounds, Prover–Delayer games, Pigeonhole principle

## Abstract

We investigate the proof complexity of modal resolution systems developed by Nalon and Dixon (J Algorithms 62(3–4):117–134, 2007) and Nalon et al. (in: Automated reasoning with analytic Tableaux and related methods—24th international conference, (TABLEAUX’15), pp 185–200, 2015), which form the basis of modal theorem proving (Nalon et al., in: Proceedings of the twenty-sixth international joint conference on artificial intelligence (IJCAI’17), pp 4919–4923, 2017). We complement these calculi by a new tighter variant and show that proofs can be efficiently translated between all these variants, meaning that the calculi are equivalent from a proof complexity perspective. We then develop the first lower bound technique for modal resolution using Prover–Delayer games, which can be used to establish “genuine” modal lower bounds for size of dag-like modal resolution proofs. We illustrate the technique by devising a new modal pigeonhole principle, which we demonstrate to require exponential-size proofs in modal resolution. Finally, we compare modal resolution to the modal Frege systems of Hrubeš (Ann Pure Appl Log 157(2–3):194–205, 2009) and obtain a “genuinely” modal separation.

## Introduction

The central problem in proof complexity is to determine the size of the smallest proof for a given formula in a specified proof system, typically defined through a set of axioms and inference rules. Since its inception, proof complexity has enjoyed close links to computational complexity [31] through the aim of separating complexity classes (sometimes referred to as Cook’s programme [29]) and to first-order logic through the tight correspondence between proof systems and theories of bounded-arithmetic (cf. [8, 30, 44]).

Recently, one of the major motivations in proof complexity has been its close connection to SAT solving [29, 54]. SAT solvers have turned into ubiquitous tools for the solution of hard computational problems from almost all application domains [46], yet a theoretical understanding of their effectiveness is only initially developed. The main approach comes through proof complexity. The trace of the run of a SAT solver on an unsatisfiable formula can be interpreted as a proof of unsatisfiability, whereby each solver implicitly defines a proof system for unsatisfiable formulas. Modern SAT solvers using conflict-driven clause learning (CDCL) correspond to propositional resolution in this sense [5, 55]. Thus, understanding the complexity of resolution refutations directly relates to the performance of SAT solvers. In particular, lower bounds on the size of resolution proofs correspond to lower bounds on the running time of SAT solvers.

In the last decade, the success of SAT solving has been transferred to more powerful logics. In particular, there has been a surge of research on quantified Boolean formulas (QBF), both in terms of solving [22, 41, 45] and in a proof complexity analysis of their associated proof systems [10, 12, 14].

This paper focuses on modal logic and their resolution calculi. Modal logics play a key role in computer science as they provide increased expressive flexibility for many application scenarios. This has led to a wealth of modal logics, including, e.g. the important temporal and description logics. Consequently, a vast number of logical calculi exist for different modal logics [36], in terms of resolution calculi [1, 35, 48–50], Frege systems [40, 42], sequent systems [62] or tableaux calculi [37]. Many of these—and in particular the modal resolution system [50]—have also been used as the underlying principle for implementations, giving rise to efficient algorithms for automated theorem proving for these logics [47, 49, 51, 59].

It is therefore rather surprising that there is only comparatively little work on the proof complexity of these systems. Probably the most important proof complexity results on modal logics concern exponential lower bounds for modal Frege systems [40, 42]. From a proof complexity perspective, Frege systems are quite strong calculi comprised of logical axioms and rules [31]. To show lower bounds for propositional Frege constitutes a major open problem [6]. At first sight, it might therefore seem rather surprising that [40, 42] obtain unconditional and exponential lower bounds for modal Frege, which augment propositional Frege with extra rules. However, the lower bounds in [40, 42] are obtained for modal formulas and actually give a lower bound purely on the number of modal steps. This already shows that proof complexity of modal logics can present a rather different picture in comparison to propositional proof complexity (cf. also [23] for a discussion).

In contrast to these exciting results on modal Frege systems, to the best of our knowledge, there has been no research whatsoever on the proof complexity of modal resolution systems. This is in stark contrast to the propositional setting where the main bulk of research has been devoted to propositional resolution and its variants [60]. As mentioned above, understanding proof size in resolution is the main avenue towards a complexity analysis of solvers, both for SAT [54] as well as for resolution-based modal provers [51].

In this work, we aim to initiate a proof complexity analysis of modal resolution systems. Our main contributions can be summarised as follows:

**1. Comparing modal resolution systems** We start with reviewing the modal resolution systems of Nalon and Dixon [48] and Nalon et al. [50]. These systems build on propositional resolution and augment it by several rules allowing to perform resolution on modal pivots. The calculi work on suitable normal forms (called separated normal forms) and come in two variants: the basic calculus $\mathbf{K }_n$-Res of [48] and the “layered” modal resolution system $\mathbf{K }_{ml}$-Res of [50]. The latter is a restricted version of the former. In $\mathbf{K }_{ml}$-Res, each clause is equipped with a label, indicating the modal level of a clause, i.e. the number of modalities in which scope the clause is. Resolution steps are only allowed in $\mathbf{K }_{ml}$-Res between clauses of matching modal levels. This calculus was introduced in [50] to streamline derivations and result in better modal provers [51].

Here we introduce one further restriction of $\mathbf{K }_{ml}$-Res, where we not only record the modal level of clauses, but the actual modal context, i.e. the precise sequence of modalities $\Box _{a_i}$ and $\Diamond _{a_j}$ (as we work in a multimodal setting). In our new calculus $\mathbf{K }_{mc}$-Res, resolution can only be performed between clauses with “unifiable” modal contexts (Definition 13). We show that $\mathbf{K }_{mc}$-Res is sound and complete (Theorem 3).

We then use the standard proof complexity concept of simulations [31] to show that proof size in all three calculi $\mathbf{K }_n$-Res, $\mathbf{K }_{ml}$-Res, and $\mathbf{K }_{mc}$-Res is polynomially related and moreover, proofs can be efficiently translated between the three systems (Theorem 4). This mainly boils down to showing that $\mathbf{K }_{mc}$-Res p-simulates $\mathbf{K }_n$-Res, i.e. we show that sub-derivations allowed in $\mathbf{K }_n$-Res but forbidden in $\mathbf{K }_{mc}$-Res are not useful and can be pruned from the proofs. The other simulations of $\mathbf{K }_{mc}$-Res by $\mathbf{K }_{ml}$-Res and $\mathbf{K }_{ml}$-Res by $\mathbf{K }_n$-Res follow by definition as the systems extend each other. Thus, from a proof complexity standpoint, all the three systems are equivalent. This has the advantage that when aiming at lower bounds we can concentrate on the streamlined system $\mathbf{K }_{mc}$-Res.

**2. A game-based lower bound technique for modal resolution** Most research effort in proof complexity is directed towards showing lower bounds for the proof size of specific families of formulas. Arguably, what is even more important than the actual lower bounds is to obtain *generally applicable lower bound techniques*. While a host of techniques is available for propositional resolution [6, 60], we here devise the first such technique for modal resolution.

Our technique uses the idea of a game between a Prover, who wants to establish the unsatisfiability of the formula, and a Delayer who claims to know a model and aims to play consistently as long as possible. In the course of the game, the Prover poses questions on the structure of the purported model. The Delayer does not answer this question himself, instead deferring the choice to the Prover and earning some points proportional to the progress that Prover makes towards a contradiction.

We can then show that if Delayer has a strategy to earn at least *n* points (where *n* is an integer) on a modal formula $\phi$ (in any game against every possible Prover), then each refutation of $\phi$ in $\mathbf{K }_{mc}$-Res has size at least $2^n$ (Theorem 5). For this, we devise modal decision trees (Definition 25), which represent partial Kripke models. These modal decision trees correspond to the partial models constructed during a game, and the size of modal decision trees is proportional to the number of modal resolution steps in $\mathbf{K }_{mc}$-Res refutations (Proposition 4). The lower bound then follows as the Delayer’s score provides a lower bound for the logarithm of the size of the modal decision trees.

Our game is inspired by similar Prover–Delayer games for propositional resolution [18, 19, 57] and QBF [17]. However, there are crucial differences: in the propositional and QBF settings, Prover asks for values of propositional variables, whereas here we do not ask for variable assignments, but for branchings in Kripke frames. This implies (via the corresponding notions of decision trees) that the propositional and QBF games measure the size of *tree-like* resolution proofs, where derived clauses may not be reused in proofs. Thus, the propositional games only provide lower bounds for the weaker tree-like model. In contrast, our game here yields lower bounds for the unrestricted *dag-like* model of proofs, which in the propositional case is known to admit exponentially shorter proofs [27].

**3. An exponential lower bound for modal resolution** We illustrate the lower bound method on a new family of formulas, which we call the modal pigeonhole principle (MPHP, Definition 26). These formulas express the classical pigeonhole principle (PHP). However, in contrast to the well-known propositional encoding, our formulas only encode the holes through propositional variables, while the pigeons are encoded through the accessibility relation in the Kripke frames.

Devising a suitable Delayer strategy that scores $\log n!$ points, we show that proofs of MPHP in modal resolution require not just exponential size, but indeed proofs of size *n*! in the dag-like model. This is in contrast to the propositional proof complexity of PHP, which is known to be $2^{\Omega (n \log n)}$ in tree-like [32], but only $2^{\Omega (n)}$ in dag-like propositional resolution [38].

We also highlight that our game only counts the number of modal resolution steps, but ignores propositional resolution steps. Thus, the lower bound shown here is a “genuine” modal lower bound and not just a lifted propositional hardness result.^1^

**4. Comparing modal resolution to modal Frege** Finally, we compare the modal resolution systems considered here to the modal Frege system of [40]. As is to be expected, we confirm that modal Frege p-simulates modal resolution. Using an argument similar to [28], we show that the modal pigeonhole principle becomes easy in modal Frege, thus providing an exponential separation. Of course, such a separation already follows from the separation of the propositional fragments (resolution vs Frege [28, 38]). However, we are interested here in a genuinely modal separation that only counts modal inferences, and MPHP are the first formulas to provide this.

In this connection, it is interesting to mention the modal clique-colour formulas investigated by Hrubeš and shown to be hard in modal Frege [40]. While these formulas are still hard for modal resolution—and hence do not separate modal resolution and modal Frege—we note that their hardness for modal resolution cannot be shown via our game technique. Thus, in contrast to the case of propositional and QBF resolution, where the asymmetric Prover–Delayer game is known to precisely characterise tree-like resolution size [17, 18], our game here does not provide a similar characterisation in the modal setting.

**Organisation** The paper is organised as follows. In Sect. 2, we give a brief overview of modal logic (for a full introduction, see [25]) and proof complexity. In Sect. 3, we review the two clausal modal resolution systems $\mathbf{K }_n$-Res [48] and $\mathbf{K }_{ml}$-Res [50], which we complement in Sect. 4 by a new proof systems $\mathbf{K }_{mc}$-Res. In Sect. 5, we show that $\mathbf{K }_n$-Res, $\mathbf{K }_{ml}$-Res, and $\mathbf{K }_{mc}$-Res are all equivalent in terms of proof complexity. In Sect. 6, we develop our lower bound technique through games, which we apply in Sect. 7 to show the hardness of the new modal pigeonhole formulas for modal resolution. Finally, we compare modal resolution and modal Frege in Sect. 8 and conclude with a discussion in Sect. 9.

## Preliminaries

### Modal Logics

A *multimodal logic* over some finite set of agents ${\mathscr {A}}=\{a_1,\dots ,a_n\}$ is an extension of propositional logic (PL) constructed from a set of propositional variables, ${\mathscr {P}}=\{p_1, p_2,\dots \}$, a complete set of propositional connectives $\{\lnot ,\wedge ,\vee \}$, the constants 0 and 1, and a set of unary modal operators $\{\Box _{a_i} \mid a_i\in {\mathscr {A}}\}$. Further, we define $\rightarrow$ so that $\phi \rightarrow \psi \equiv \lnot \phi \vee \psi$ and for each $i\in [n]$ (where [*n*] denotes the set $\{1,\dots ,n\}$) and we define the modal operator $\Diamond _{a_i}\equiv \lnot \Box _{a_i}\lnot$. Throughout the paper we will take $\circ _a\in \{\Box _a,\Diamond _a\}$. The formulas $\Box _a\phi$ and $\Diamond _a\phi$ are read as “agent *a* considers $\phi$ to be necessary” and “agent *a* considers $\phi$ to be possible”, respectively. A full introduction to modal logics is given in [25].

A *literal* is either a propositional variable, $p\in {\mathscr {P}}$, or its negation, $\lnot p$. We let ${\mathscr {L}}$ denote the set of all literals. A *positive modal literal* (resp. *negative modal literal*) is a formula of the form $\Box _al$ (resp. $\Diamond _al$), where $a\in {\mathscr {A}}$ and $l\in {\mathscr {L}}$. A *modal literal* is a positive or negative modal literal. A *clause* is a disjunction of literals. The empty disjunction is referred to as the *empty clause*. We let $\mathscr {CL}$ denote the set of all clauses. We say a formula is in *conjunctive normal form* (CNF) if it is a conjunction of clauses.

Let ${\mathscr {P}}$ be a set of propositional variables and ${\mathscr {A}}$ be a finite set of agents. We define the set of *well-formed multimodal formulas*, denoted wfmf, to be the smallest possible set s.t. $1\in \text {wfmf}$,$0\in \text {wfmf}$, $p\in \text {wfmf}$ for all $p\in {\mathscr {P}}$ and $\phi \in \text {wfmf}$ if either $\phi =\lnot \psi$, $\phi =(\psi \wedge \theta )$, $\phi =(\psi \vee \theta )$ or $\phi =\Box _a \psi$ where $\psi , \theta \in \text {wfmf}$ and $a\in {\mathscr {A}}$.

The multimodal logic $\mathbf{K }_n$ is the smallest set that contains all propositional tautologies, all formulas of the form $\text {K}_a : \Box _a(\phi \rightarrow \psi )\rightarrow (\Box _a \phi \rightarrow \Box _a \psi )$ and is closed under the inference rules modus ponens $\frac{\phi \quad \phi \rightarrow \psi }{\psi }$ and *a*-necessitation $\frac{\phi }{\Box _a\phi }$ for every $a\in {\mathscr {A}}$.

The semantics of multimodal logics are given using Kripke models. A *Kripke model* (henceforth a model) over a set of propositional variables ${\mathscr {P}}$ and a set of agents ${\mathscr {A}}=\{a_1,\dots , a_n\}$ is a tuple $M=(W, R_{a_1},\dots ,R_{a_n},V)$, where *W* is a non-empty set of “worlds”, each $R_{a_i}$ is a binary relation over *W*, which we call the $a_i$-accessibility relation, and *V* is a set of valuation functions $\{V(w) \mid w\in W\}$ s.t. $V(w): {\mathscr {P}}\mapsto \{0,1\}$.

A *pointed model* is a pair $\langle M,w\rangle$ consisting of a Kripke model $M=(W, R_{a_1},\dots ,R_{a_n}, V)$ together with some distinguished world $w\in W$.

We say that a model $M'=(W',R_{a_1}',\dots , R_{a_n}', V')$ *extends* a model $M=(W,R_{a_1},\dots , R_{a_n}, V)$ if and only if $X\subseteq X'$ for all $X\in \{W\}\cup \{R_{a_i}\mid i\in [n]\}\cup \{V\}$. Further, $V'(w)(x)=V(w)(x)$ for every $w\in W\cap W'$ and every *x* in the domain of *V*(*w*).

Let $\phi ,\psi$ be formulas and $p\in {\mathscr {P}}$. Given a model $M=( W, R_1,\dots R_n, V)$ and a world $w\in W$ the *satisfiability* of a formula at *w* in *M* is defined inductively as follows:

- $$(M,w)\models p \iff w\in V(p)$$,
- $(M,w)\models \lnot \phi \iff (M,w)\models \phi$ does not hold (written as $(M,w)\not \models \phi$)
- $$(M,w)\models \phi \wedge \psi \iff (M,w)\models \phi $$ and $$(M,w)\models \psi $$,
- $$(M,w)\models \phi \vee \psi \iff (M,w)\models \phi $$ or $$(M,w)\models \psi $$,
- $$(M,w)\models \Box _a\phi \iff (M,w')\models \phi $$ for all $$w'$$ s.t. $$(w,w')\in R_a$$.

We say $\phi$ is *satisfiable* if there exists some $w_0\in W$ s.t. $(M, w_0)\models \phi$. We say that a pointed model $\langle M,w\rangle$ satisfies $\phi$ iff $(M,w)\models \phi$.

For two modal formulas $\phi$ and $\psi$, we write $\phi \models \psi$ if for every (*M*, *w*) as above, $(M,w) \models \phi$ implies $(M,w)\models \psi$.

### Proof Complexity

#### Definition 1

[31] A *proof system* for some language $L\subseteq \Sigma ^*$ is a polynomial-time computable partial function $P:\Sigma ^*\rightarrow L$ where $\Sigma ^*$ denotes the set of all finite words over $\Sigma$. A *P proof* of some $\tau \in L$ is a word $\pi \in \Sigma ^*$ s.t. $P(\pi )=\tau$.

The above definition of a proof system is rather general. In this paper we will only consider *line-based* proof systems. A *line-based proof system* is a proof system defined by some finite set of inference rules and axioms. A *proof* in a line-based proof system is a sequence of proof lines, say $\lambda _1,\dots , \lambda _n$ s.t. each $\lambda _i$ is either an axiom of *P* or can be inferred by applying some rule of *P* to some subset of $\{\lambda _1,\dots , \lambda _{i-1}\}$.

#### Definition 2

Let *P* be a line-based proof system and let $\pi$ be a *P* proof. Further, let R be some inference rule of *P* and let $\lambda _1$ and $\lambda _2$ be lines of $\pi$. Then, $\lambda _2$ is a *child* (an *R child*) of $\lambda _1$ if it is inferred by applying an inference rule (R) to a set of lines containing $\lambda _1$.

We say that $\lambda _2$ is a *descendant* (an *R descendant*) of $\lambda _1$ if it is either:

(i) a child (an R child) of $\lambda _1$ or,
(ii) a child (an R child) of a descendant (an R descendant) of $\lambda _1$.

If $\lambda _2$ is a descendant (an R descendant) of $\lambda _1$ then $\lambda _1$ is an *ancestor* (an *R ancestor*) of $\lambda _2$.

Let *P* be a proof system. We say that $\psi$ is *P* *provable* from $\phi$ if there exists a *P* proof of $\psi$ from $\phi$. We denote this by $\phi \vdash _P\psi$. We say *P* is *strongly complete* if for all formulas $\phi ,\psi$ s.t. $\phi \models \psi$ we have $\phi \vdash _P \psi$. Further, we say a proof system is *complete* if for every $\phi$ s.t. $\phi \models 0$ we have $\phi \vdash _P 0$. We say *P* is *sound* if for every formula $\phi$ s.t. $\vdash \phi$ we have $\models \phi$. The *size* of a proof $\pi$ is the number of symbols it contains, denoted $|\pi |$.

We can compare the strength of two proof systems for a given language *L* using polynomial simulations.

#### Definition 3

[31] Let *P* and *Q* be *L*-proof systems. We say that *P* *polynomially simulates* (p-simulates) *Q*, denoted $Q\le _p P$, if there exists a polynomial time computable function *f* s.t. for any *Q* proof $\pi$ we have $P(f(\pi ))=Q(\pi )$.

We say that *P* and *Q* are *polynomially equivalent* (p-equivalent) if $P\le _p Q$ and $Q\le _p P$, denoted $P\equiv _p Q$.

### Propositional Resolution

Resolution is a simple proof system for propositional logic [26, 33, 58]. It acts on formulas in CNF (defined in Sect. 2.1) and consists of the single rule:

$$\begin{aligned} \hbox {RES:} \frac{C_1\vee l \quad C_2\vee \lnot l}{C_1\vee C_2} \end{aligned}$$

where $C_1, C_2$ are clauses and *l* is a literal. The intuition behind this rule is straightforward. No propositional model can simultaneously satisfy a literal and its negation, hence if we take the disjunct of any two clauses containing complementary literals we may “cut away” (resolve on) said complementary literals. Throughout we will refer to the variable resolved on as a *pivot* variable.

Resolution is a *refutational* proof system. This means that to prove that a formula is valid^2^ using resolution we prove that its negation is unsatisfiable. So to prove that some formula $\phi$ is valid we would first convert its negation into CNF and then repeatedly apply the resolution rule until we derive the empty clause which is logically equivalent to 0.

## Modal Resolution Systems

Constructing a resolution-based proof system for even the basic multimodal logic $\mathbf{K }_n$ is not straightforward. This is because whether or not we can only resolve complementary literals with one another now depends on the “modal context” in which they occur. To see this, consider the formulas $\phi =\Box _{a_1} (l_1\vee l_2\vee l_3)$, $\psi =\lnot l_1\vee l_2$, $\theta =\Box _{a_1} \lnot l_2$ and $\zeta = \Diamond _{a_1} \lnot l_3$. In any sound and complete $\mathbf{K }_n$ resolution system the following three statements should be true:

1. The instance of $l_1$ in $\phi$ cannot be resolved with the instance of $\lnot l_1$ in $\psi$.
2. The instance of $l_2$ in $\phi$ can be resolved with the instance of $\lnot l_2$ in $\theta$ to obtain a resolvent of the form $\Box _{a_1}(l_1\vee l_3)$.
3. The instance of $l_3$ in $\phi$ can be resolved with the instance of $\lnot l_3$ in $\zeta$ to obtain a resolvent of the form $\Diamond _{a_1} (\lnot l_3\wedge (l_1\vee l_2))$.

Statement 1 is true as the instance of $l_1$ in $\phi$ is nested within the scope of a $\Box _{a_1}$ operator whereas the instance of $\lnot l_1$ in $\psi$ is not within the scope of any modal operator.

Statement 2 holds as the instance of $\lnot l_2$ in $\phi$ and the instance of $\lnot l_2$ in $\theta$ are both nested within a single $\Box _{a_1}$. Hence, it follows that if $\phi$ and $\theta$ are both satisfied at some world *w* in some model $M=(W,R_1,\dots ,R_n,V)$ then $l_1\vee l_2\vee l_3$ and $\lnot l_2$ must both be satisfied at every world $w_1$ s.t. $(w,w_1)\in R_1$ and so $l_1\vee l_3$ must also be satisfied at every $w_1$.

Finally, the instance of $l_3$ in $\phi$ appears within the scope of a $\Box _{a_1}$ operator and the instance of $\lnot l_3$ in $\zeta$ appears within the scope of a $\Diamond _{a_1}$ operator. Hence, if $\phi$ and $\zeta$ are both satisfied at some world *w* in some model $M=(W,R_1,\dots ,R_n,V)$ then $l_1\vee l_2\vee l_3$ must be satisfied at every world $w_1\in W$ s.t. $(w,w_1)\in R_1$ and $\lnot l_3$ must be satisfied at some world $w_2\in W$ s.t. $(w,w_2)\in R_1$. And so it follows by classical resolution that $l_1\vee l_2$ must also be satisfied at $w_2$, hence statement 3 holds.

As a result of this added complexity, several different $\mathbf{K }_n$ resolution systems have been proposed. In this section, we shall review two such clausal resolution systems. These systems, which we shall refer to as $\mathbf{K }_n$-Res and $\mathbf{K }_{ml}$-Res, are closely related to each other and were proposed by Nalon and Dixon [48], and Nalon et al. [50], respectively.

### The Proof System $\mathbf{K }_n$-Res

Nalon and Dixon [48] proposed a clausal resolution system for $\mathbf{K }_n$, which we shall call $\mathbf{K }_n$-Res. This proof system determines whether a formula $\phi$ is satisfiable at some distinguished “start” world, $s_0\in W$. However, as the choice of $s_0$ is arbitrary, determining the satisfiability of $\phi$ at $s_0$ is essentially equivalent to determining the satisfiability of $\phi$.

Let $M=( W, R_1,\dots , R_n, V)$ be a model and $w_1,$ $w_2\in W$. We say $w_{2}$ is *reachable* from $w_1$ if $(w_1,w_2)$ is in the reflexive and transitive closure of $\bigcup _{i=1}^nR_i$. Note that every world is reachable from itself. We define the *master modality*, denoted $\Box ^*$, s.t. $(M,w)\models \Box ^*\phi$ iff $(M, w')\models \phi$ for all $w'$ reachable from *w*.

The proof system $\mathbf{K }_{n}$-Res operates on formulas that have been translated into the following normal form.

#### Definition 4

[48] Let $l, l',l_j\in {\mathscr {L}}$ and let ${\mathbf {S}}$ be a nullary connective defined s.t. $(M,w)\models {\mathbf {S}}$ iff $w=s_0$. A formula $\phi$ is in *separated normal form* ($\text {SNF}$) if $\phi =\bigwedge _{i=1}^r \Box ^* C_i$ where each $C_i$ is of one of the following types:
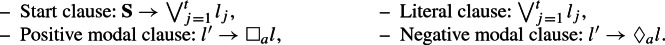


#### Definition 5

[48] Any $\phi \in \text {wfmf}$ in *negation normal form* (NNF)^3^ can be translated into a set of $\text {SNF}$ clauses by applying the function:

$$\begin{aligned} \tau _0(\phi )=\Box ^*({\mathbf {S}}\rightarrow x_{\varepsilon })\wedge \tau _1(\Box ^*(x_{\varepsilon }\rightarrow \phi )), \end{aligned}$$

where $x_{\varepsilon }$ is a new variable and the function $\tau _1$ is defined as follows:

$$\begin{aligned} \tau _1(\Box ^* (x\rightarrow \phi \wedge \psi ))&= \tau _1(\Box ^* (x\rightarrow \phi ))\wedge \tau _1(\Box ^*(x\rightarrow \psi )).&\\ \tau _1(\Box ^* (x\rightarrow \circ _a \phi ))&={\left\{ \begin{array}{ll} \Box ^* (x\rightarrow \circ _a \phi ), &{} \text {if } \phi \in {\mathscr {L}},\\ \Box ^* (x\rightarrow \circ _a x_1)\wedge \tau _1(\Box ^* (x_1\rightarrow \phi )), &{} \text {otherwise.} \end{array}\right. }&\\ \tau _1(\Box ^* (x\rightarrow \phi \vee \psi ))&={\left\{ \begin{array}{ll} \Box ^* (\lnot x\vee \phi \vee \psi ), &{} \text {if } \phi ,\psi \in \mathscr {CL},\\ \begin{array}{l} \Box ^* (\lnot x\vee x_1\vee x_2)\wedge \tau _1(\Box ^*(x_1\rightarrow \phi ))\\ \qquad \wedge \tau _1 (\Box ^* (x_2\rightarrow \psi )), \end{array} &{}\text {if }\phi ,\psi \not \in \mathscr {CL}, \\ \Box ^* (\lnot x\vee \phi \vee x_1)\wedge \tau _1(\Box ^* (x_1\rightarrow \psi )), &{} \text {if }\phi \in \mathscr {CL}, \psi \not \in \mathscr {CL}.\\ \end{array}\right. }&\end{aligned}$$

where $x_1$ and $x_2$ are new variables. Note that in the disjunctive transformation $\psi$ may be the empty clause, in which case the usual simplification rules are applied at the end of the transformation.

We refer to the variables introduced when translating a formula $\phi \in \text {wfmf}$ into a set of SNF clauses ${\mathscr {C}}$ as *extension variables* and define ${\mathscr {X}}_{{\mathscr {C}}}$ to be the set of all such variables. Further, we define ${\mathscr {X}}_{{\mathscr {C}}+}=\{x'\in {\mathscr {X}}\mid \Box ^*(x\rightarrow \Box _a x')\in {\mathscr {C}}\}$, ${\mathscr {X}}_{{\mathscr {C}}-}=\{x'\in {\mathscr {X}}\mid \Box ^*(x\rightarrow \Diamond _a x')\in {\mathscr {C}}\}$ and ${\mathscr {X}}_{{\mathscr {C}}\pm }={\mathscr {X}}_{{\mathscr {C}}+}\cup {\mathscr {X}}_{{\mathscr {C}}-}$. Note that ${\mathscr {X}}_{{\mathscr {C}}}\subseteq {\mathscr {L}}$.

Let ${\mathscr {C}}$ be a set of SNF clauses and let $C\in {\mathscr {C}}$. We say $x\in {\mathscr {X}}_{{\mathscr {C}}}$ *appears positively* in *C* if either *C* is a literal clause of the form $\Box ^*(x\vee D)$ where $D\in \mathscr {CL}$ or *C* is a modal clause of the form $(x'\rightarrow \circ _a x)$ where $x'\in {\mathscr {X}}_{{\mathscr {C}}}$. We say *x* *appears negatively* in *C* if either *C* is a literal clause of the form $\Box ^*(\lnot x\vee D)$ or *C* is a modal clause of the form $(x\rightarrow \circ _a y)$.

#### Example 1

Consider the modal formula $\phi = (x \vee \Diamond _a \lnot y )\wedge \Box _a y \wedge \lnot x$. Then,

$$\begin{aligned}\tau _0(\phi )&= \Box ^*({\mathbf {S}} \rightarrow x_{0}) \wedge \tau _1( x_{0} \rightarrow \phi )\\&= \Box ^*({\mathbf {S}} \rightarrow x_{0}) \wedge \tau _1(\Box ^*( x_{0} \rightarrow (x \vee \Diamond _a \lnot y))) \wedge \tau _1( \Box ^*(x_{0} \rightarrow \Box _a y)) \wedge \tau _1( \Box ^*(x_{0} \rightarrow \lnot x)) \\&= \Box ^*({\mathbf {S}} \rightarrow x_{0}) \wedge \tau _1(\Box ^*( x_{0} \rightarrow x_1 \vee x_2)) \wedge \tau _1(\Box ^*( x_{1} \rightarrow x )) \; \wedge \\&\qquad \qquad \qquad \qquad \tau _1( \Box ^*(x_2 \rightarrow \Diamond _a \lnot y)) \wedge \Box ^*(x_{0} \rightarrow \Box _a y) \wedge \Box ^*(\lnot x_{0} \vee \lnot x)\\&= \Box ^*({\mathbf {S}} \rightarrow x_{0}) \wedge \Box ^*( \lnot x_{0} \vee x_1 \vee x_2) \;\wedge \\&\qquad \qquad \qquad \qquad \Box ^*(\lnot x_{1} \vee x ) \wedge \Box ^*(x_2 \rightarrow \Diamond _a \lnot y) \wedge \Box ^*(x_{0} \rightarrow \Box _a y) \wedge \Box ^*(\lnot x_{0} \vee \lnot x). \end{aligned}$$

Further, $${\mathscr {X}}_{\tau _0(\phi )} = \{x_{0}, x_1, x_2\}$$ and $${\mathscr {X}}_{\tau _0(\phi )+} = {\mathscr {X}}_{\tau _0(\phi )-}= \emptyset $$.

A proof that $\tau _0$ preserves satisfiability is given in [48].

As every SNF clause is prefixed by $\Box ^*$ it follows that every SNF clause occurs within the same modal context. Hence, the inference rules of $\mathbf{K }_n$-Res are relatively straightforward.

#### Definition 6

**Fig. 1:**
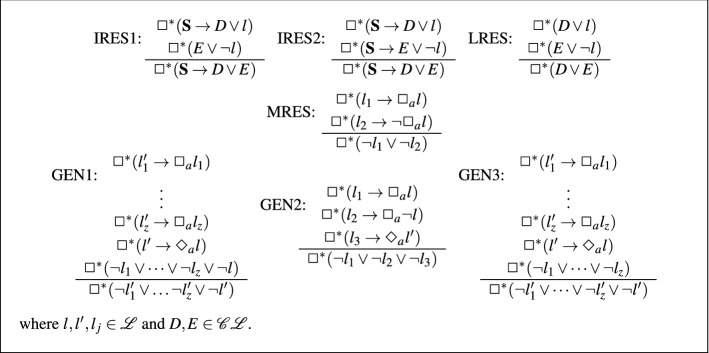
Rules for $\mathbf{K }_n$-Res

[48] The inference rules of **K**$_n$-Res are given in Fig. 1.

The rules of $\mathbf{K }_n$-Res can be split into two categories, modal rules (GEN1, GEN2, and GEN3) and propositional rules (MRES, LRES, IRES1, and IRES2). Each of the modal rules is used to resolve on literals inside some modal operator.

The rules IRES1, IRES2, LRES and MRES are essentially propositional resolution and are each used to resolve a formula with its negation. The rule GEN2 says that if we have some negative modal clause, say $\Box ^*(l_3'\rightarrow \Diamond _a l_2)$, then we can resolve two positive modal literals of the form $\Box _a l_1$ and $\Box _a \lnot l_1$ with one another. The negative modal clause is required for soundness as $\Box ^*(l_1'\rightarrow \Box _a l_1)$ and $\Box ^*(l_2'\rightarrow \Box _a\lnot l_1)$ can both be satisfied by a model *M* at a world $w\in W$ s.t. $(w,w')\notin R_a$ for all $w'\in W$.

The rules GEN1 and GEN3 resolve literals with modal literals. More specifically, GEN1 says that given some clause $\Box ^*(\lnot l_1\vee \dots \vee \lnot l_z\vee \lnot l)$ we can simultaneously resolve the $z+1$ literals $\lnot l_1,\dots , \lnot l_z$ and $\lnot l$ with the modal literals $\Box _a l_1,\dots , \Box _a l_z$ and $\Diamond _a l$. When resolving literals with modal literals in this way we are taking advantage of the fact that, by the definition of $\Box ^*$, any world in any model which satisfies $\Box ^*(\lnot l_1\vee \dots \vee \lnot l_z\vee \lnot l)$ must also satisfy $\Box _a(\lnot l_1\vee \dots \vee \lnot l_z\vee \lnot l)$. Every literal in $\Box ^*(\lnot l_1\vee \dots \vee \lnot l_z \vee \lnot l)$ must be resolved on simultaneously as otherwise the resolvent obtained may not be in SNF. For example the resolvent obtained by resolving $\lnot l_1$ in $\Box _a(\lnot l_1\vee \dots \vee \lnot l_z\vee \lnot l)$ with $\Box _a l_1$ in $\Box ^*(l_1'\rightarrow \Box _a l_1)$ would be $\Box ^*(\lnot l_1'\vee \Box _a(l_2\vee \dots \vee \lnot l_z\vee \lnot l))$. The rule GEN3 is similar to GEN1, however the negative modal literal, $\Diamond _a\lnot l$, is not resolved on. Instead, as in the case for GEN2, it is necessary only for soundness.

Like propositional resolution, the proof system $\mathbf{K }_n$-Res is a refutational system. Let $\pi$ be a $\mathbf{K }_n$-Res refutation of some set of clauses ${\mathscr {C}}$ and let *C* be some clause in ${\mathscr {C}}$. If $C\in {\mathscr {C}}$ then we say that *C* is an *initial clause* (also sometimes called input clause). If $C\not \in {\mathscr {C}}$ then we say *C* is a *non-initial* clause.

#### Example 2

Let $\phi$ be defined as in Example 1. Further, let ${\mathscr {C}}$ be the set of SNF clauses obtained by applying *T* to $\phi$. We can refute ${\mathscr {C}}$ using $\mathbf{K }_n$-Res as follows:
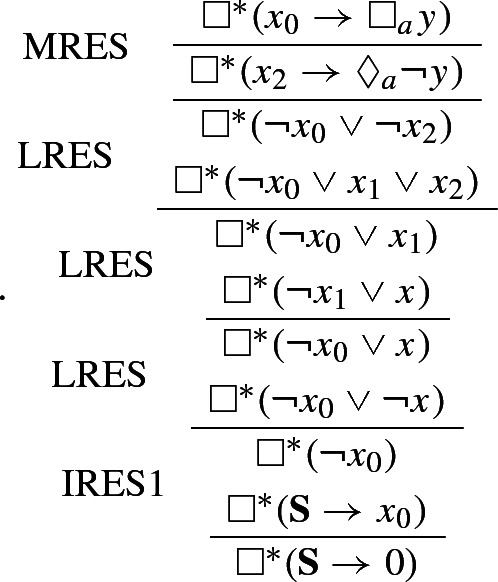


### The Proof System **K**$_{ml}$-Res

In [50] Nalon, Hustadt and Dixon introduced a layered resolution system for $\mathbf{K }_n$ which we shall call $\mathbf{K }_{ml}$-Res. This resolution system is similar to $\mathbf{K }_n$-Res, however it operates on a normal form where each clause is labelled by its modal level. The *modal level* of a clause is the number of modal operators it was nested within in the original formula. I note that the full $\mathbf{K }_{ml}$-Res calculus presented in [50] can be used for both local and global reasoning, however here are concerned with, and hence present, only the local resolution part of the system.

#### Definition 7

[50] A formula $\phi$ is in *separated normal form with modal levels* ($\hbox {SNF}_{{ml}}$) if $\phi =\bigwedge _{i=1}^rC_i$ where each clause $C_i$ is either a: 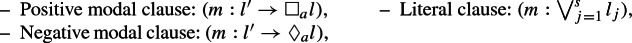


where $l, l',l_j\in {\mathscr {L}}$ and $m\in \mathbb {N}$ representing the modal level of the clause.

Let $M=(W,R_1,\dots , R_n, V)$ be a Kripke model and $w,w'\in W$. We say $w'$ is of *distance* *m* from *w* if there exists a path of length *m* from *w* to $w'$ through the union of all accessibility relations in *M*.

The satisfiability of some $\phi \in \text {wfmf}$ labelled by its modal level, $m\in \mathbb {N}$, at root world $w_0$ is given as follows:

$$\begin{aligned} (M, w_0)\models (m : \phi )\iff (M,w)\models \phi \text { for all } w\in W \text { s.t. } w \text { is of distance } m \text { from } w_0\in W. \end{aligned}$$

The following procedure for efficiently translating any NNF formula into $\hbox {SNF}_{{ml}}$ $\phi$, while preserving satisfiability, is given in [50]. To convert an NNF formula $\phi$ into $\hbox {SNF}_{{ml}}$ we apply the translation $T_{ml}(\phi )= (0:x) \wedge \rho _{ml}(0: x \rightarrow \phi )$, where *x* is a new propositional variable and $\rho _{ml}$ is defined as follows:

$$\begin{aligned}&\rho _{ml}(m: x\rightarrow \theta \wedge \psi )=\rho _{ml}(m: x\rightarrow \theta )\wedge \rho _{ml}(m:x\rightarrow \psi ),\\&\rho _{ml}(m: x\rightarrow \Box _a \theta )\;\;\;={\left\{ \begin{array}{ll} (m:x\rightarrow \circ _a \theta ), &{} \text {if } \theta \in {\mathscr {L}},\\ (m: x\rightarrow \circ _a x_1)\wedge \rho _{ml}(m+1: x_1\rightarrow \theta ), &{} \text {otherwise.} \end{array}\right. }\\&\rho _{ml}(m:x\rightarrow \theta \vee \psi ))={\left\{ \begin{array}{ll} (m:\lnot x\vee \theta \vee \psi ), &{} \text {if } \theta ,\psi \in \mathscr {CL},\\ \rho _{ml}(m:x\rightarrow \theta \vee x_1)\wedge \rho _{ml}(m: x_1\rightarrow \psi ), &{} \text {otherwise,} \end{array}\right. } \end{aligned}$$

where $\theta , \psi$ are formulas, $x_1$ is a new propositional symbol and $m\in \mathbb {N}$. Note that $\rho _{ml}(m: x\rightarrow \theta \vee x_1)=\rho _{ml}(m: x\rightarrow (x_1\vee x_2))\wedge \rho _{ml}(m:x_2\rightarrow \theta )$. Note also that $\theta$ or $\psi$ could be empty in which case simplification of the formulas applies.

#### Example 3

Let $$\phi = (x \vee \Diamond _a (\lnot y \wedge x)) \wedge \Box _a y \wedge \lnot x$$. Then,

$$\begin{aligned} T_{ml}(\phi )&= (0 : x_{0}) \wedge (0 : \lnot x_0 \vee x_1 \vee x_2) \wedge ( 0 : \lnot x_1 \vee x) \wedge (0 : x_2 \rightarrow \Diamond _a x_3) \,\wedge \\&\qquad \qquad \quad (1 : \lnot x_3 \vee \lnot y) \wedge (1 : \lnot x_3 \vee x) \wedge (0 : x_0 \rightarrow \Box _a y) \wedge (0 : \lnot x_0 \vee \lnot x). \end{aligned}$$

#### Theorem 1

[50] An NNF formula $\phi$ is satisfiable iff $T_{ml}(\phi )$ is satisfiable.

#### Definition 8

**Fig. 2:**
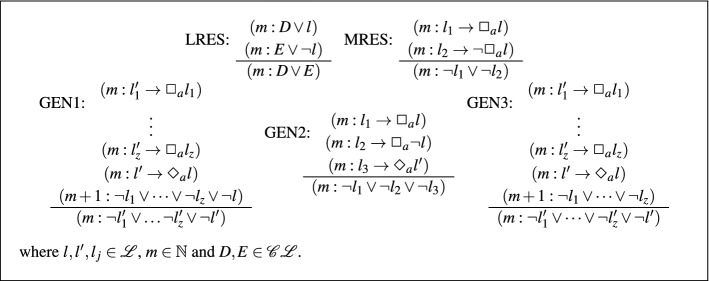
Rules for $\mathbf{K }_{ml}$-Res

[50] The inference rules of **K**$_{ml}$-Res are given in Fig. 2.

The rules of $\mathbf{K }_{ml}$-Res are almost identical to those of $\mathbf{K }_n$-Res, however now LRES, MRES, and GEN2 may only be applied to clauses that are at the same modal level. Further, GEN1 and GEN3 may only be applied to sets of clauses where each modal clause is the same modal level and the literal clause is at the modal level above. The rules IRES1 and IRES2 are no longer necessary as we are no longer determining satisfiability at a fixed start world.

We shall see in Sect. 5 that the proof systems $\mathbf{K }_{ml}$-Res and $\mathbf{K }_{n}$-Res are equivalent in terms of proof complexity.

## Resolution with Modal Contexts

In this section we will define a new modal resolution system called $\mathbf{K }_{mc}$-Res. The rules of this proof system are essentially identical to those of $\mathbf{K }_{ml}$-Res, however it acts on a normal form where each clause is labelled by its *modal context* as opposed to its modal level.

Informally, if we give each $\Diamond$ operator in some modal formula $\phi$ a unique label then the modal context of a subformula $\psi$ of $\phi$ is the sequence of modal operators that it is nested within in $\phi$. So for example if $\phi _1=\Box _a\Diamond _{a'}^1 x\wedge \Box _a\Diamond _{a'}^2y$ then *x* has modal context $\Box _a \Diamond _{a'}^1$ and *y* has modal context $\Box _a \Diamond _{a'}^2$. Whereas if $\phi _2=\Diamond _a^1(x\wedge y)$ then both *x* and *y* have modal context $\Diamond _a^1$. Intuitively two subformulas of $\phi$ have the same modal context iff in any model of $\phi$ these subformulas must be evaluated at exactly the same world or worlds. Clearly there exist models that satisfy $\phi _1$ but do not contain any world *w* s.t. $V(w)(x)=1$ and $V(w)(y)=1$, however every model that satisfies $\phi _2$ contains a world where $V(w)(x)=V(w)(y)=1$. Hence, in our new calculus $\mathbf{K }_{mc}$-Res we label each clause by its modal context to help determine which clauses can be resolved together.

To refute a formula using $\mathbf{K }_{mc}$-Res we must first translate it into a clausal form, where each clauses modal context w.r.t. the original formula is explicitly given. As the translation used introduces a new unique extension variable for every complex subformula of the form $\Diamond _a\phi$ where $\phi \not \in {\mathscr {L}}$ we do not need to label the $\Diamond _a$ operators. The modal context of a clause can be specified by a finite word over the set of agents and the set of pairs of the form (*a*, *x*) where *a* is an agent and *x* is an extension variable.

### Definition 9

Let $l, l', l_j\in {\mathscr {L}}$. A formula $\phi$ is in *separated normal form with modal contexts* ($\hbox {SNF}_{{mc}}$) if $\phi =\bigwedge _{i=1}^rC_i$ where each $C_i$ is either a: 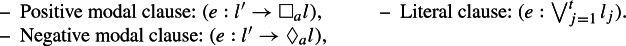


Here, *e* is a finite word over ${\mathscr {E}}_{{\mathscr {C}}}$ (Definition 10) denoting the modal context of the clause.

To convert an NNF formula $\phi$ into $\hbox {SNF}_{{mc}}$ we apply the translation $T_{mc}(\phi )= x_{\varepsilon }\wedge \rho _{mc}(\varepsilon : x_{\varepsilon } \rightarrow \phi )$, where *x* is a new propositional variable and $\rho _{mc}$ is defined as follows:

$$\begin{aligned}&\rho _{mc}(e: x\rightarrow \theta \wedge \psi )=\rho _{mc}(e: x\rightarrow \theta )\wedge \rho _{mc}(e:x\rightarrow \psi ),\\&\rho _{mc}(e: x\rightarrow \Box _a \theta )\;\;\;={\left\{ \begin{array}{ll} (e:x\rightarrow \Box _a \theta ), &{} \text {if } \theta \in {\mathscr {L}},\\ (e: x\rightarrow \Box _a x_1)\wedge \rho _{mc}(ea: x_1\rightarrow \theta ), &{} \text {otherwise.} \end{array}\right. }\\&\rho _{mc}(e: x\rightarrow \Diamond _a \theta )\;\;\;={\left\{ \begin{array}{ll} (e: x\rightarrow \Diamond _a \theta ), &{} \text {if } \theta \in {\mathscr {L}},\\ (e: x\rightarrow \Diamond _a x_1)\wedge \rho _{mc}(e(a, x_1): x_1\rightarrow \theta ), &{} \text {otherwise.} \end{array}\right. }\\&\rho _{mc}(e:x\rightarrow \theta \vee \psi ))={\left\{ \begin{array}{ll} (e:\lnot x\vee \theta \vee \psi ), &{} \text {if } \theta ,\psi \in \mathscr {CL},\\ \rho _{mc}(e:x\rightarrow \theta \vee x_1)\wedge \rho _{mc}(e: x_1\rightarrow \psi ), &{} \text {otherwise,} \end{array}\right. } \end{aligned}$$

where $\theta , \psi$ are formulas, $x_1$ is a new propositional symbol and $e\in (\{x_{\varepsilon }\}\cup {\mathscr {A}}\cup ({\mathscr {A}} \times {\mathscr {X}}_{{\mathscr {C}}-}))^*$. Note that $\rho _{mc}(e: x\rightarrow \theta \vee x_1)=\rho _{mc}(e: x\rightarrow (x_1\vee x_2))\wedge \rho _{mc}(e:x_2\rightarrow \theta )$. Also, $\theta$ or $\psi$ could be empty in which case simplification of the formulas applies.

### Example 4

Let $$\phi = (x \vee \Diamond _a (\lnot y \wedge x)) \wedge \Box _a y \wedge \lnot x$$. Then,

$$\begin{aligned} T_{mc}(\phi ) =&(\varepsilon : x_{0}) \wedge (\varepsilon : \lnot x_0 \vee x_1 \vee x_2) \wedge (\varepsilon : \lnot x_1 \vee x) \wedge ( \varepsilon : x_2 \rightarrow \Diamond _a x_3) \,\wedge \\&( (a, x_3) : \lnot x_3 \vee \lnot y) \wedge ( (a, x_3) : \lnot x_3 \vee x) \wedge ( \varepsilon : x_0 \rightarrow \Box _a y) \wedge ( \varepsilon : \lnot x_0 \vee \lnot x). \end{aligned}$$

Let ${\mathscr {C}}$ be a set of $\hbox {SNF}_{{mc}}$ clauses inferred by applying $\rho _{mc}$ to some formula $\phi \in \text {wfmf}$. As in Sects. 3.1 and 3.2 we refer to the variables added during the translation as extension variables and define the sets ${\mathscr {X}}_{{\mathscr {C}}}$, ${\mathscr {X}}_{{\mathscr {C}}-}$, ${\mathscr {X}}_{{\mathscr {C}}+}$ and ${\mathscr {X}}_{{\mathscr {C}}\pm }$ in the obvious way.

### Definition 10

For any set of $\hbox {SNF}_{{mc}}$ clauses ${\mathscr {C}}$, we define the set of clausal modal context markers to be:

$$\begin{aligned} {\mathscr {E}}_{{\mathscr {C}}} = {\mathscr {A}}\cup ({\mathscr {A}}\times {\mathscr {X}}_{{\mathscr {C}}-}). \end{aligned}$$

The set of all finite words over ${\mathscr {E}}_{{\mathscr {C}}}$ (denoted ${\mathscr {E}}_{{\mathscr {C}}}^*$) then consists of all modal contexts for ${\mathscr {C}}$.

The length of a modal context $e\in {\mathscr {E}}_{{\mathscr {C}}}^*$ is denoted |*e*| and is defined as follows:

$$\begin{aligned} |e|={\left\{ \begin{array}{ll} 0, &{} \text {if } e=\varepsilon ,\\ |e'|+1, &{} \text {if } e=e'c \text { for some } e'\in {\mathscr {E}}_{{\mathscr {C}}}^*, c\in {\mathscr {A}}\cup ({\mathscr {A}}\times {\mathscr {X}}_{{\mathscr {C}}-}). \end{array}\right. } \end{aligned}$$

Intuitively, each label $(a,x)\in {\mathscr {A}}\times {\mathscr {X}}_{{\mathscr {C}}-}$ refers to the unique $\Diamond _a$ operator s.t. $(e: x'\rightarrow \Diamond _a x)\in {\mathscr {C}}$. That this $\Diamond _a$ is unique follows from the definition of the translation $T_{mc}$ as each extension variable in ${\mathscr {X}}_{{\mathscr {C}}-}$ appears exactly once as a modal literal. Each label $a\in {\mathscr {A}}$ refers to a $\Box _a$ operator.

### Remark 1

As the set of modal context markers for a set of $\hbox {SNF}_{{mc}}$ clauses is defined in terms of the extension variables introduced by the translation function (as well as the agents), throughout this paper we consider only sets of $\hbox {SNF}_{{mc}}$ clauses that have been generated using this function, and our results are restricted to such sets of $\hbox {SNF}_{{mc}}$. Indeed, the resolution system does not make much sense when applied to sets of $\hbox {SNF}_{{mc}}$ clauses that have not been generated in such a manner.

### Definition 11

Let $M=(W,R_{a_1},\dots , R_{a_n}, V)$ be a Kripke model, let $\phi \in \text {wfmf}$ and let ${\mathscr {C}}=T_{mc}(\phi )$. We say $w\in W$ is $\varepsilon$ reachable from $w_{\varepsilon }\in W$ if $w=w_{\varepsilon }$. We say *w* is *ea*-reachable from $w_{\varepsilon }$ if $(w',w)\in R_a$ for some $w'\in W$ s.t. $w'$ is *e*-reachable from *w*. We say *w* is *e*(*a*, *x*)-reachable from $w_{\varepsilon }$ where $x\in {\mathscr {X}}_{{\mathscr {C}}-}$ and $a\in {\mathscr {A}}$ if $(w',w)\in R_a$ for some $w'\in W$ s.t. $w'$ is *e*-reachable from *w* and $V(w)(x)=1$.

We define the satisfiability of a clause with modal context $e\in {\mathscr {E}}_{{\mathscr {C}}}^*$ at root world $w_{\varepsilon }$ as follows:

$$\begin{aligned} (M,w_{\varepsilon })\models (e: C) \iff (M,w)\models C \text { for all } w\in W \text { s.t. } w \text { is }e\text {-reachable from }w_{\varepsilon }\in W. \end{aligned}$$

### Definition 12

Let ${\mathscr {C}}$ be a set of $\hbox {SNF}_{{mc}}$ clauses and $x'\in {\mathscr {X}}_{{\mathscr {C}}}$. We say that $x'$ is *propositionally reachable* from $x\in {\mathscr {X}}_{{\mathscr {C}}\pm }$ if either $x=x'$ or there exists some subset of ${\mathscr {C}}$:

$$\begin{aligned} {\mathscr {C}}_{(x,x')}=\{(e: D_1\vee \lnot x_1\vee x_2),\dots ,(e:D_n\vee \lnot x_{n-1}\vee x_n)\} \end{aligned}$$

where $x_1=x$, $x_n=x'$, $x_i\in {\mathscr {X}}_{{\mathscr {C}}}$ for each $i\in [n]$ and each $D_i\in \mathscr {CL}$. We say that such a set ${\mathscr {C}}_{(x,x')}$ *witnesses* that $x'$ is propositionally reachable from *x*.

It follows immediately from the above definition and the definition of $T_{mc}$ that every variable $x'\in {\mathscr {X}}_{{\mathscr {C}}}$ is propositionally reachable from some unique $x\in {\mathscr {X}}_{{\mathscr {C}}\pm }$. Further, the set $C_{(x,x')}$ witnessing this is unique.

### Theorem 2

An NNF formula $\phi$ is satisfiable if and only if $T_{mc}(\phi )$ is satisfiable.

### Proof

By Theorem 1, an NNF formula $\phi$ is satisfiable iff the set of $\hbox {SNF}_{{ml}}$ clauses $T_{ml}(\phi )$ is satisfiable. Hence, we prove the theorem by showing that $T_{ml}(\phi )$ is satisfiable iff $T_{mc}(\phi )$ is.

It follows immediately from the definitions of $T_{ml}$ and $T_{mc}$ that there is a one-to-one correspondence between the set of $\hbox {SNF}_{{ml}}$ clauses $T_{ml}(\phi )$ and the set of $\hbox {SNF}_{{mc}}$ clauses $T_{mc}(\phi )$. That is, $(m: C)\in {\mathscr {C}}_{ml}$ iff $(e: C)\in {\mathscr {C}}_{mc}$ for some $e\in {\mathscr {E}}_{{\mathscr {C}}}^*$ s.t. $|e|=m$.

($\Rightarrow$:) Suppose ${\mathscr {C}}_{ml}$ is satisfiable. Then, there exists some model $M=(W,R_{1},\dots , R_n,V)$ and some $w_{\varepsilon }\in W$ s.t. $(M,w_{\varepsilon })\models (m: C)$ for every $(m:C)\in {\mathscr {C}}_{ml}$. It follows by definition that $(M, w)\models C$ for all $w\in W$ s.t. *w* is of distance *m* from $w_{\varepsilon }$. Every world that is *e*-reachable from $w_{\varepsilon }$, where $|e|=m$ must also be of distance *m* from $w_{\varepsilon }$ hence $(M, w_e)\models C$ for all $w_e\in W$ s.t. $w_e$ is *e*-reachable from $w_{\varepsilon }$ and so $(M,w_{\varepsilon })\models (e: C)$ for all $e\in {\mathscr {E}}_{{\mathscr {C}}}^*$ s.t. $|e|=m$. Hence, $(M,w_{\varepsilon })\models {\mathscr {C}}_{mc}$.

($\Leftarrow$:) Now suppose the set ${\mathscr {C}}_{mc}$ is satisfiable. Let $M=(W,R_{1},\dots , R_n,V)$ be a model that satisfies ${\mathscr {C}}_{mc}$ at $w_{\varepsilon }\in W$. Suppose $(e:C)\in {\mathscr {C}}_{mc}$ where $e\in {\mathscr {E}}_{{\mathscr {C}}}^*$ and let $|e|=m$. We will show that $(M,w_{\varepsilon })\models (m: C)$ via induction on |*e*|.

Suppose $|e|=0$, then $e=\varepsilon$. Clearly $w_{\varepsilon }$ is the only world in *W* that is distance 0 from itself. Further, $w_{\varepsilon }$ is the only world in *W* that is $\varepsilon$-reachable from itself. By assumption $(M,w_{\varepsilon })\models (\varepsilon : C)$, hence it follows that $(M,w_{\varepsilon })\models C$ and so $(M,w_{\varepsilon })\models (0: C)$.

Suppose $|e|>0$. By the definition of $T_{mc}$, the clause *C* must contain exactly one negative extension literal *x*. Further, *x* must be propositionally reachable from some unique $x'\in {\mathscr {X}}_{{\mathscr {C}}\pm }$. We prove by induction on the size of the set witnessing this that we can assume w.l.o.g. that $V(w)(x)=0$ for all $w\in W$ s.t. *w* is not *e*-reachable from $w_{\varepsilon }$.

If $|C_{(x',x)}|=1$, then $x\in {\mathscr {X}}_{{\mathscr {C}}\pm }$. Suppose $x\in {\mathscr {X}}_{{\mathscr {C}}-}$ ($x\in {\mathscr {X}}_{{\mathscr {C}}+}$). It follows from the definition of $T_{mc}$ that $e=e'(a,x)$ ($e=e'a$) and $(e':x'\rightarrow \Diamond _a x)\in {\mathscr {C}}_{mc}$ ($(e':x'\rightarrow \Box _a x)\in {\mathscr {C}}_{mc}$). Further, by the definition of $T_{mc}$ this is the only clause containing *x* positively and every clause containing *x* negatively has modal context *e*. Hence, we can assume w.l.o.g. that $V(w)(x)=0$ for all $w\in W$ s.t. *w* is not *e*-reachable from $w_{\varepsilon }$. As *C* contains $\lnot x$ it is satisfied at every such *w*. Hence, $(M,w_{\varepsilon })\models (m: C)$.

Finally suppose $|C_{(x',x)}|=k$. Then, by definition $C_{(x',x)}$ must contain some clause $C'$ of the form $(\lnot x_1\vee D\vee x)$ where $x_1\in {\mathscr {X}}_{{\mathscr {C}}}$. Further, $C_{(x',x)}{\setminus }\{C'\}$ witnesses that $x_1$ is propositionally reachable from $x'$. Hence, by induction $V(w)(x_1)=0$ at all worlds *w* that are not *e*-reachable from $x_{\varepsilon }$. It follows that we can assume w.l.o.g. that $x=0$ at every such world as doing so will not effect the truth valuation $C'$ which is the only clause containing the positive literal *x*. Hence, *C* is satisfied at every world that is not *e*-reachable from $w_{\varepsilon }$. Further, by assumption *C* is satisfied at every world that is *e*-reachable from $w_{\varepsilon }$ and so $(M,w_{\varepsilon })\models (m:C)$.$\square$

In our new calculus, we allow inferences to be made from sets of clauses with different modal contexts under certain conditions. To see why this is necessary, consider the formula $\phi =\Box _a (x\wedge y)\wedge \Diamond _a (\lnot x\wedge z)$ and the corresponding set of $\hbox {SNF}_{{mc}}$ clauses ${\mathscr {C}}=\{(\varepsilon :x_{\varepsilon }),\,(\varepsilon :x_{\varepsilon }\rightarrow \Box _a x_1),\,(a:\lnot x_1\vee x),\,(a:\lnot x_1\vee y),\,(\varepsilon :x_{\varepsilon }\rightarrow \Diamond _a x_2),\, ((a,x_2):\lnot x_2\vee \lnot x),\, ((a,x_2):\lnot x_2\vee z)\}$. Clearly $\phi$ is unsatisfiable, however we cannot refute ${\mathscr {C}}$ using similar rules to those of $\mathbf{K }_{n}$-Res and $\mathbf{K }_{ml}$-Res if we do not allow inferences on clauses with different modal contexts. Hence, we have the following definition.

### Definition 13

Let ${\mathscr {C}}$ be a set of $\hbox {SNF}_{{mc}}$ clauses. We define the function $\sigma :{\mathscr {E}}_{{\mathscr {C}}}^*\times \dots \times {\mathscr {E}}_{{\mathscr {C}}}^* \mapsto {\mathscr {E}}_{{\mathscr {C}}}^*$ so that $\sigma (\varepsilon ,\dots , \varepsilon )= \varepsilon$, for $c_1,\dots , c_n\in {\mathscr {E}}_{{\mathscr {C}}}$:

$$\begin{aligned} \sigma (c_1,\dots ,c_n)={\left\{ \begin{array}{ll} c_j &{} \text {if } c_j=(a,x)\in {\mathscr {A}}\times {\mathscr {X}}_{{\mathscr {C}}} \text { and } c_k\in \{a,c_j\} \text { for all } k\ne j,\\ a &{} \text {if } c_1=\dots =c_n=a\in {\mathscr {A}},\\ \text {undefined} &{} \text {otherwise,} \end{array}\right. } \end{aligned}$$

and for $$e_1,\dots e_n\in {\mathscr {E}}_{{\mathscr {C}}}^*$$:

$$\begin{aligned} \sigma (e_1,\dots , e_n)={\left\{ \begin{array}{ll} \sigma (c_{1,1},\dots ,c_{1,n})\dots \sigma (c_{m,1},\dots , c_{m,n}) &{} \text {if } |e_1|=\dots =|e_n|=m>0,\\ \text {undefined} &{} \text {otherwise,} \end{array}\right. } \end{aligned}$$

where $c_{i,j}$ denotes the *i*th letter in the word $e_j$.

We say that the modal contexts $e_1,\dots , e_n\in {\mathscr {E}}_{{\mathscr {C}}}^*$ are *unifiable* if $\sigma (e_1,\dots , e_n)$ is defined. Otherwise we say that $e_1,\dots ,e_n$ are *non-unifiable*.

Intuitively allowing resolution on sets of clauses with unifiable modal contexts can be thought of as allowing $\Box _a$ to be resolved with $\Diamond _a$ to infer $\Diamond _a$, which is of course sound.

### Definition 14

**Fig. 3:**
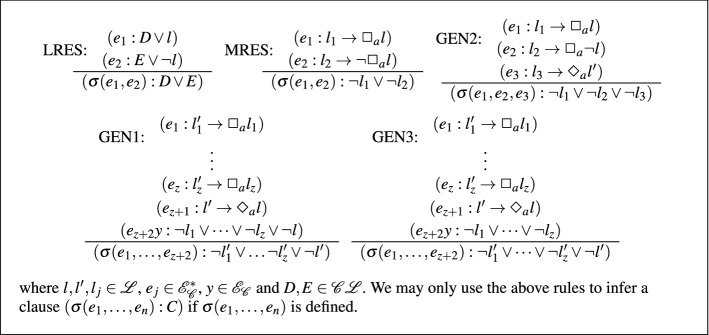
Rules for $\mathbf{K }_{mc}$-Res

The inference rules of **K**$_{mc}$-Res are given in Fig. 3.

### Remark 2

Let ${\mathscr {C}}$ be a set of $\hbox {SNF}_{{mc}}$ clauses and let *C* be some clause which is $\mathbf{K }_{mc}$-Res derivable from ${\mathscr {C}}$. If *C* has modal context $e\in {\mathscr {E}}^*_{{\mathscr {C}}}$ then it must be inferred by applying some rule of $\mathbf{K }_{mc}$-Res to a set of clauses, whose modal contexts are either unifiable with *e* or unifiable with *ec* for some $c\in {\mathscr {E}}^*_{{\mathscr {C}}}$. It follows by induction that *C* is $\mathbf{K }_{mc}$-Res derivable from the subset of ${\mathscr {C}}$ consisting of every clause whose modal context is a suffix of some $e'\in {\mathscr {E}}^*_{{\mathscr {C}}}$ which is unifiable with *e*.

### Theorem 3

$\mathbf{K }_{mc}$-Res is sound and is complete.

### Proof

Clearly, any proof system that simulates a complete proof system is complete and any proof system that is simulated by a sound proof system is sound. We will show simulations between all the modal resolution systems $\mathbf{K }_n$-Res, $\mathbf{K }_{ml}$-Res and $\mathbf{K }_{mc}$-Res in the next section, leading to Theorem 4. As $\mathbf{K }_n$-Res is sound and complete [48], the theorem then immediately follows from Theorem 4. $\square$

Note that we could prove this theorem directly by following a very similar method to the one used in [50] to show that $\mathbf{K }_{ml}$-Res is complete.

## Comparing $\mathbf{K }_{n}$-Res, $\mathbf{K }_{ml}$-Res, and $\mathbf{K }_{mc}$-Res

In this section, we prove that the modal resolution systems $\mathbf{K }_n$-Res, $\mathbf{K }_{ml}$-Res and $\mathbf{K }_{mc}$-Res are polynomially equivalent. That $\mathbf{K }_{mc}\text {-Res} \le _p\mathbf{K }_{ml}\text {-Res}\le _p\mathbf{K }_n$-Res follows straightforwardly from the respective definitions of the proof systems. To prove that $\mathbf{K }_{n}$-$\text {Res}\le _p\mathbf{K }_{ml}\text {-Res}\le _p\mathbf{K }_{mc}$-Res, we show that given an unsatisfiable set of SNF clauses, ${\mathscr {C}}$ and a $\mathbf{K }_{n}$-Res refutation of ${\mathscr {C}}$, $\pi$ the following statement is true. The sequence of clauses obtained from $\pi$ by deleting every clause inferred from a set of clauses whose modal contexts would prevent the analogous rule of $\mathbf{K }_{mc}$-Res from being applied to ${\mathscr {C}}'$, along with every descendant of such a clause, is also a ${\mathbf {K}}_{n}$-Res refutation of ${\mathscr {C}}$. So for example if $\pi$ contains a clause *C* that was inferred by applying LRES to two literal clauses with modal contexts $e_1$ and $e_2$, respectively, and $\sigma (e_1,e_2)$ is undefined then $\pi '$ would not contain *C* or any descendant of *C*.

### Modal Contexts for Clauses in SNF

To prove that $\mathbf{K }_{mc}\text {-Res}\le _p\mathbf{K }_{ml}\text {-Res}\le _p\mathbf{K }_{n}\text {-Res}$ we must be able to “read off” the modal context of a given clause in SNF. In Sect. 4, we saw that the extension variables introduced when translating a modal formula into $\hbox {SNF}_{{mc}}$ encode the modal context of each clause. This is also true of the extension variables introduced when translating a modal formula into SNF. Hence, in this subsection we give a series of definitions that enable us determine the modal context of a clause simply by looking at the extension variables it contains.

In Sect. 4, we defined what it meant for an extension variable $x'\in {\mathscr {X}}_{{\mathscr {C}}}$ to be propositionally reachable from some $x\in {\mathscr {X}}_{{\mathscr {C}}\pm }$ for a given set of $\hbox {SNF}_{{mc}}$ clauses ${\mathscr {C}}$. Similarly, for any set of SNF clauses ${\mathscr {C}}$ we say that $x'\in {\mathscr {X}}_{{\mathscr {C}}}$ is propositionally reachable from some $x\in {\mathscr {X}}_{{\mathscr {C}}\pm }$ if there exists some subset of ${\mathscr {C}}$ of the form:

$$\begin{aligned} {\mathscr {C}}_{(x,x')}= \{\Box ^*(x_0\rightarrow \circ _a x_1),\Box ^*(D_1\vee \lnot x_1\vee x_2),\dots ,\Box ^*(D_n\vee \lnot x_{n-1}\vee x_n)\}, \end{aligned}$$

where $x_1=x$ and $x_{n}=x'$. We further define the set $E_{{\mathscr {C}}}$ so that for every $x_1,x_2\in {\mathscr {X}}$ we have $(x_1,x_2)\in E_{{\mathscr {C}}}$ iff $x_2$ is propositionally reachable from $x_1$.

#### Definition 15

Let $y\in {\mathscr {X}}_{{\mathscr {C}}}$. We say that *y* is *a*-*positively modally reachable* (resp. *a*-*negatively modally reachable*) from *x* if ${\mathscr {C}}$ contains a clause of the form $\Box ^*(x' \rightarrow \Box _a y')$ (resp. $\Box ^*(x' \rightarrow \Diamond _a y')$) where $x',y'\in {\mathscr {X}}_{{\mathscr {C}}}$, $(x,x')\in E_{{\mathscr {C}}}$ and $(y',y)\in E_{{\mathscr {C}}}$.

We define $E_{{\mathscr {C}}}^{a+}$ (resp. $E_{{\mathscr {C}}}^{a-}$) so that $(x,y)\in E^{a+}_{{\mathscr {C}}}$ (resp. $(x,y)\in E^{a-}_{{\mathscr {C}}}$) iff *y* is *a*-positively modally reachable (resp. *a*-negatively modally reachable) from *x*.

Let ${\mathscr {C}}$ be a set of SNF clauses. We can specify the modal context of a given extension variable in ${\mathscr {X}}_{{\mathscr {C}}}$ or clause in ${\mathscr {C}}$ using finite words over the set ${\mathscr {E}}_{{\mathscr {C}}}$ (Definition 10).

#### Definition 16

Let ${\mathscr {C}}$ be a set of SNF clauses. We define:

$$\begin{aligned} {\mathscr {X}}_{x_{\varepsilon }}=\{x\in {\mathscr {X}}_{{\mathscr {C}}}: (x_{\varepsilon },x)\in E_{{\mathscr {C}}}\}. \end{aligned}$$

For every $e\in {\mathscr {E}}_{{\mathscr {C}}}^*$ and every $c\in {\mathscr {E}}_{{\mathscr {C}}}$ we define:

$$\begin{aligned} {\mathscr {X}}_{ec}={\left\{ \begin{array}{ll} \{x\in {\mathscr {X}}_{{\mathscr {C}}}: (z,x)\in E_{{\mathscr {C}}}^{a+},\; z\in {\mathscr {X}}_{e}\}&{} \text {if } c\in {\mathscr {A}},\\ \{x\in {\mathscr {X}}_{{\mathscr {C}}}: (x,z)\in E_{{\mathscr {C}}}\} &{} \text {if } c=(a,z) \text { for some } z\in {\mathscr {X}}_{{\mathscr {C}}-} \text { and } a\in {\mathscr {A}},\\ \emptyset &{} \text {otherwise.} \end{array}\right. } \end{aligned}$$

We say *x* has *modal context* *e* if $x\in {\mathscr {X}}_e$.

#### Definition 17

Let $\phi$ be a well-formed modal formula in NNF and let ${\mathscr {C}}=\tau _0(\phi )$ be the set of SNF clauses generated by applying the translation function $\tau _0$ to $\phi$. Further, let $\pi$ be a $\mathbf{K }_{n}$-Res refutation of ${\mathscr {C}}$ and let *C* be some clause in $\pi$. If *C* contains $\mathbf{S }$ then we say that *C* has *modal context* $x_{\varepsilon }$. Further, if some $x\in {\mathscr {X}}_{e}$ appears as a negative literal (*not* a negative modal literal) in *C* the we say that *C* has *modal context* *e*.

Let ${\mathscr {C}}$ be a set of SNF clauses generated by applying the translation function $\tau _0$ to some well-formed modal formula $\phi$ in NNF. It follows from the definition of $\tau _0$ that each initial clause $C\in {\mathscr {C}}$ contains only one negative extension literal, and so each such *C* has only one modal context. However, using the rules of $\mathbf{K }_n$-Res it is possible to derive non-initial SNF clauses that contain two or more negative extension literals with distinct modal contexts and so have multiple modal contexts. We will make use of the modal contexts of SNF clauses in our proof that $\mathbf{K }_n$-Res is polynomially simulated by $\mathbf{K }_{mc}$-Res in the next section.

### The Polynomial Simulations

In this subsection we give a proof that $\mathbf{K }_{n}$-Res, $\mathbf{K }_{ml}$-Res and $\mathbf{K }_{mc}$-Res are all p-equivalent. Proving that $\mathbf{K }_{mc}\text {-Res}\le _p\mathbf{K }_{ml}\text {-Res} \le _p\mathbf{K }_{n}$-Res is trivial. Hence, the majority of the subsection is made up of a series of lemmas that are used to prove that $\mathbf{K }_{n}\text {-Res}\le _p\mathbf{K }_{ml}\text {-Res} \le _p\mathbf{K }_{mc}$-Res. We begin by giving some definitions and results concerning the structure of $\mathbf{K }_n$-Res proofs.

#### Definition 18

Let $\pi$ be a $\mathbf{K }_n$-Res refutation of some set of SNF clauses ${\mathscr {C}}$, let ${\mathscr {C}}_{\pi }$ denote the set of all clauses in $\pi$ and let $C_1, C_n\in {\mathscr {C}}_{\pi }$. We say that there is a *path* from $C_1$ to $C_n$ if there exists a word $C_1\dots C_n\in \mathscr {C_{\pi }}^{*}$ s.t. for each $i\in [n-1]$ the clause $C_{i+1}$ is a child of $C_i$.

#### Lemma 1

Let ${\mathscr {C}}$ be a set of clauses in SNF and $\pi$ be a $\mathbf{K }_{n}$-Res refutation of ${\mathscr {C}}$. If $C_2=\Box ^*(x\vee D_2)$ is a descendant of $C_1=\Box ^*(x\vee D_1)\in {\mathscr {C}}$, where $x\in {\mathscr {X}}_{{\mathscr {C}}}$ then $\pi$ contains a path *P* from $C_1$ to $C_2$ s.t. every clause in *P* contains *x*.

#### Proof

As $C_2$ is a descendant of $C_1$ the refutation $\pi$ contains a path $P_1=A_1\dots A_n$ where $C_1=A_1$, $C_2=A_n$. Let *S* be the longest suffix of $P_1$ s.t. every $A_j\in S$ contains *x*. We proceed by induction on the size of *S*. If $|S|=|\pi |$ then as $|S|\le |P_1|\le |\pi |$ it follows that $S=P_1$.

Suppose $|S|<|\pi |$ then either $S=P_1$ or $S=A_{j},\dots , A_n$ where $j>1$. In the latter case $x\not \in A_{j-1}$ and so $A_{j}$ must be also a child of some $C'\ne A_{j}$ where either $C'=C_1$ or $C'$ is a descendant of $C_1$ containing *x*. Hence, there exists a path $P_2$ from $C_1$ to $A_{j}$. Concatenating $P_2$ with $A_{j+1},\dots , A_{n}$ gives a path from $C_1$ to $C_2$ with a suffix of length $\ge |S|+1$. Hence, by the inductive hypothesis there exists a path *P* from $C_1$ to $C_2$ s.t. every clause in *P* contains *x*. $\square$

#### Definition 19

We say a $\mathbf{K }_n$-Res refutation:

$$\begin{aligned} \pi =C_1,\dots , C_{n-1}, C_n=\Box ^*({\mathbf {S}}\rightarrow 0), \end{aligned}$$

is in *1-start form* if it contains precisely two start clauses, namely $C_n$ and some $C_j=\Box ^*({\mathbf {S}}\rightarrow x_0)$ where $j\in [n-1]$. Equivalently, we say $\pi$ is in 1-start form if it does not contain any clauses inferred using IRES2 and $C_n$ is the only clause in $\pi$ inferred using IRES1.

#### Proposition 1

Let ${\mathscr {C}}$ be an unsatisfiable set of SNF clauses and $\pi =C_1,\dots , C_n$ be a **K**$_{n}$-Res refutation of ${\mathscr {C}}$. From $\pi$ we can efficiently construct a 1-start refutation of ${\mathscr {C}}$ with size $\le |\pi |$.

#### Proof

If $\pi$ is in 1-start form then the proposition holds trivially. Hence, we suppose $\pi$ is not is 1-start form and proceed to construct a new refutation as follows. First we delete from $\pi$ every clause that is inferred by applying IRES1 to $\Box ^*(\mathbf{S }\rightarrow x_{\varepsilon })$ and some literal clause $\ne \Box ^*(\lnot x_{\varepsilon })$. Let ${\mathscr {S}}=\{S_1,\dots , S_m\}$, where each $S_i$ is of the form $\Box ^*(\mathbf{S }\rightarrow D_i)$, be the set of all remaining non-initial start clauses in $\pi$. Replacing each $S_i$ in $\pi$ with $\Box ^*(\lnot x_{\varepsilon }\vee D_i)$ yields a derivation of $\Box ^*(\lnot x_{\varepsilon })$, hence by adding the clauses $\Box ^*(\mathbf{S }\rightarrow x_{\varepsilon })$ and $\Box ^*(\mathbf{S }\rightarrow 0)$ to the end of $\pi$ we obtain a valid refutation of ${\mathscr {C}}$ in 1-start form. $\square$

Hence, from this point onwards we will consider only $\mathbf{K }_n$-Res refutations in 1-start form.

We will now prove three technical lemmas. Let $\pi$ be a $\mathbf{K }_n$-Res refutation of some unsatisfiable set of SNF clauses ${\mathscr {C}}$. The first of the lemmas simply states that every literal clause in $\pi$ contains at least one extension variable. The second lemma says that if a clause in $\pi$ contains some negative extension literal with modal context *e* then any clause inferred from *C* using LRES must also contain some negative extension literal with modal context *e*. The third lemma says that if a clause *C* in $\pi$ contains a negative extension literal with modal context *e* and is propositionally reachable from some $x\in {\mathscr {X}}_{{\mathscr {C}}\pm }$, then $\pi$ must also contain some clause that is an LRES descendant of *C* and contains the literal $\lnot x$.

#### Lemma 2

Let $\phi \in \text {wfmf}$, let ${\mathscr {C}}=\tau _0(\phi )$ and let $\pi$ be a $\mathbf{K }_n$-Res refutation of ${\mathscr {C}}$. Every literal clause in $\pi$ contains at least one negative extension literal.

#### Proof

We prove the lemma by induction. If $C\in {\mathscr {C}}$ then the lemma follows from the definition of $\tau _0$. Suppose *C* is inferred using some modal inference rule. By the definition of $\tau _0$ each modal clause used to infer *C* contains a negative extension literal. That *C* contains each of these literals follows by the definition of the modal rules of $\mathbf{K }_n$-Res. Suppose *C* is inferred using LRES. Let $C_1$ and $C_2$ be the clauses use to infer *C*. By the inductive hypothesis $C_1$ contains some negative extension literal $\lnot x_1$ and $C_2$ contains some negative extension literal $\lnot x_2$. Clearly $\lnot x_1$ and $\lnot x_2$ cannot be resolved with each other and so *C* must contain at least one of $\lnot x_1$ and $\lnot x_2$. $\square$

#### Lemma 3

Let $\phi \in \text {wfmf}$, let ${\mathscr {C}}=\tau _0(\phi )$ and let $\pi$ be a $\mathbf{K }_{n}$-Res refutation of ${\mathscr {C}}$ in 1-start form. Let *C* be some literal clause in $\pi$ that is inferred by applying LRES to two literal clauses $C_1=\Box ^*(\lnot y_1\vee D_1)$ and $C_2$, where $y_1\in {\mathscr {X}}_{{\mathscr {C}}}$. Let $x\in {\mathscr {X}}_{{\mathscr {C}}\pm }$ s.t. $(x,y_1)\in E_{{\mathscr {C}}}$. Then, $C=\Box ^*(\lnot y_2\vee D_2)$, where $y_2\in {\mathscr {X}}_{{\mathscr {C}}}$ s.t. $(x,y_2)\in E_{{\mathscr {C}}}$ and $|{\mathscr {C}}_{(x,y_2)}|\le |{\mathscr {C}}_{(x,y_1)}|$.

#### Proof

If $y_1$ is not the pivot variable then $C=\Box ^*(\lnot y_1\vee D_2)$ and so the lemma holds trivially. Hence, we suppose $y_1$ is the pivot variable (and hence that $C_2$ contains the literal $y_1$) and proceed by induction on $|{\mathscr {C}}_{(x,y_1)}|$.

Suppose $|{\mathscr {C}}_{(x,y_1)}|=1$. Then, ${\mathscr {C}}_{(x,y_1)}=\{C'=\Box ^*(x_0\rightarrow \circ _a x_1)\}$, where $x_1=y_1=x$. Recall that every variable in ${\mathscr {X}}_{{\mathscr {C}}}$ appears positively in exactly one clause of ${\mathscr {C}}$. Further, no clause containing the literal $y_1$ can be inferred from $C'$ using the rules of $\mathbf{K }_{n}$-Res.

Hence, $\pi$ cannot contain a literal clause containing $y_1$, contradicting our assumption that $C_2$ is such a clause.

Suppose $|{\mathscr {C}}_{(x,y_1)}|\ge 2$. The set ${\mathscr {C}}$ contains exactly one clause, say $C'=\Box ^*(\lnot y_2\vee D\vee y_1)$ in which $y_1$ appears positively. Hence, there exists a set:

$$\begin{aligned} C_{(x,y_1)}=\{\Box ^*(x_0\rightarrow \circ _a x_1),\Box ^*(\lnot x_1\vee D_1'\vee x_2),\dots , \Box ^*(\lnot x_{n-1}\vee D_{n-1}'\vee x_{n} )\}, \end{aligned}$$

where $x_1=x$, $x_{n-1}=y_2$, $x_{n}=y_1$ and $D_{n-1}'=D$. As $C_2$ contains $y_1$ and $C'$ is the only clause in ${\mathscr {C}}$ containing $y_1$ it follows that $C_2$ is a descendant of $C'$. Hence, by Lemma 1 the refutation $\pi$ must contain some path *P* from $C'$ to $C_2$ s.t. every clause in *P* contains $y_1$. Thus, no clause in *P* is inferred by resolving on $y_1$. As *P* contains no start clauses and no clauses inferred by resolving on $y_1$ each $A_j\in P$ must be inferred using LRES^4^. Clearly the set of clauses $C_{(x,y_2)}=C_{(x,y_1)}{\setminus } \{C'\}$ witnesses that $y_2$ is propositionally reachable from *x*.

We proceed to show by induction on (i) |*P*| and (ii) $|C_{(x,y_2)}|$ that $C_2$ contains some negative extension literal $\lnot y$ s.t. $(x,y)\in E_{{\mathscr {C}}}$ and $|{\mathscr {C}}_{(x,y)}|\le |{\mathscr {C}}_{(x,y_2)}|$. If $|C_{(x,y_2)}|=1$ then $y_2=x=x_1$. As in the case when $x_1=y_1$ it follows that every LRES descendant of $C'$ contains $\lnot y_2$. Suppose $|C_{(x,y_2)}|>1$. If $|P|=0$ then $C_2=C'$ and so $\lnot y_2\in C_2$. Suppose $|P|>1$ and let $P_1$ be the path from $C'$ to $C_3$ s.t. $C_2$ is a child of $C_3$. By inductive hypothesis (i) $C_3$ contains some negative extension literal $\lnot y_3$ s.t. $(x,y_3)\in E_{{\mathscr {C}}}$ and $|{\mathscr {C}}_{(x,y_3)}|\le |{\mathscr {C}}_{(x,y_2)}|$. Thus, by inductive hypothesis (ii) every descendant of $C_3$ must contain some $y_4$ s.t. $(x,y_4)\in E_{{\mathscr {C}}}$ and $|{\mathscr {C}}_{(x,y_4)}|\le |{\mathscr {C}}_{(x,y_3)}|$. In particular $C_2$ must contain some such literal.

As $|C_{(x,y_2)}|<|C_{(x,y_1)}|$ it follows that $y\ne y_1$ and so *C* must contain $\lnot y$. $\square$

#### Lemma 4

Let ${\mathscr {C}}$ be a set of SNF clauses and let $\pi$ be a $\mathbf{K }_n$-Res refutation of ${\mathscr {C}}$ in 1-start form. Suppose $\pi$ contains a literal clause $C=\Box ^*(\lnot x\vee D_1)$, where $x\in {\mathscr {X}}_{{\mathscr {C}}}$ and let $x_1\in {\mathscr {X}}_{{\mathscr {C}}\pm }$ s.t. $(x_1,x)\in E_{{\mathscr {C}}}$. If $\Box ^*(\mathbf{S }\rightarrow 0)$ is a descendant of *C* then $\pi$ contains a literal clause $C'=\Box ^*(\lnot x_1\vee E)$ s.t. $C'$ is an LRES descendant of *C* and $\Box ^*(\mathbf{S }\rightarrow 0)$ is a descendant of $C'$.

#### Proof

We will prove the lemma by induction on $|{\mathscr {C}}_{(x_1,x)}|$. If $|{\mathscr {C}}_{(x_1,x)}|=1$ then $x_1=x$ and so the lemma holds trivially.

Suppose $|{\mathscr {C}}_{(x_1,x)}|>1$. As $\Box ^*(\mathbf{S }\rightarrow 0)$ is a descendant of *C* there exists some descendant of *C* that is inferred by resolving on *x* (and possibly some other variables). Let $C'$ be the first such descendant. One of the clauses that $C'$ is inferred from must be a descendant of *C* containing $\lnot x$. Let $C_1$ denote this clause. Note that every descendant of *C* is non-initial and so $C_1$ is a literal clause. As $C'$ is inferred by resolving on *x* it must also be inferred from some clause $C_2\ne C_1$ containing the literal *x*. As $|{\mathscr {C}}_{(x_1,x)}|>1$ no modal clause in ${\mathscr {C}}$ contains the literal *x* and so $C_2$ is a literal clause. Hence, $C'$ is inferred by applying LRES to $C_1$ and $C_2$. Furthermore, as $C_1$ contains $\lnot x$ and $C'$ is the first descendant of *C* inferred by resolving on *x* it follows that $C_1$ is an LRES descendant of *C*. Hence, by Lemma 3, $C'$ is of the form $\Box ^*(\lnot x_2\vee D)$ where $x_2\in {\mathscr {X}}_{{\mathscr {C}}}$ s.t. $(x_1,x_2)\in E_{{\mathscr {C}}}$ and $|{\mathscr {C}}_{(x_1,x_2)}|<|{\mathscr {C}}_{(x_1,x)}|$. By the inductive hypothesis there exists an LRES descendant of $C'$, and so *C*, of the form $\Box ^*(\lnot x_1\vee E)$ that is also an ancestor of $\Box ^*(\mathbf{S }\rightarrow 0)$. $\square$

The following lemma is the main result of this subsection. The lemma essentially states that if a 1-start refutation of a set of SNF clauses ${\mathscr {C}}$ contains clauses having non-unifiable modal contexts, then said clauses and their descendants may be deleted from the refutation and that the resulting sequence of clauses will still be a valid refutation of ${\mathscr {C}}$. The proof of this consists of showing that any clause with non-unifiable modal contexts cannot be an ancestor of $\Box ^*({\mathbf {S}}\rightarrow 0)$ and hence can be deleted from the refutation as they are essentially “dead ends”.

#### Lemma 5

Let $\phi \in \text {wfmf}$, ${\mathscr {C}}=\tau _0(\phi )$ and $\pi$ be a $\mathbf{K }_n$-Res refutation of ${\mathscr {C}}$ in 1-start form. Let $\pi '$ be the sequence of clauses obtained by deleting some clauses $C_1,\dots , C_m$, along with every descendant of each $C_i$ from $\pi$. If each $C_i$ has non-unifiable modal contexts then $\pi '$ is a $\mathbf{K }_n$-Res refutation of ${\mathscr {C}}$.

#### Proof

Clearly any sequence $\pi '$ that is obtained from $\pi$ by removing clauses that are not ancestors of $\Box ^*(\mathbf{S }\rightarrow 0)$, as well as all of their descendants, is a refutation of ${\mathscr {C}}$. So to prove the lemma we show that $\Box ^*(\mathbf{S }\rightarrow 0)$ cannot be a descendant of any clause *C* in $\pi$ that has non-unifiable modal contexts. As all initial clauses have unifiable modal contexts any such *C* is a literal clause of the form $\Box ^*(\lnot x_1\vee \lnot x_2\vee D)$ where $D\in \mathscr {CL}$ and $x_1, x_2\in {\mathscr {X}}_{{\mathscr {C}}}$ with non-unifiable modal contexts. Let $e_1$ and $e_2$ be the modal contexts of $x_1$ and $x_2$, respectively. We assume w.l.o.g. that $|e_1|\le |e_2|$ .

Suppose *C* is an ancestor of $\Box ^*(\mathbf{S }\rightarrow 0)$. By Lemma 4 the refutation $\pi$ contains some clause $C'=\Box ^*(\lnot y_1\vee D_1)$ where $y_1\in {\mathscr {X}}_{e_1}\cap (\{x_{\varepsilon }\}\cup {\mathscr {X}}_{{\mathscr {C}}_{\pm }})$ and $D_1\in \mathscr {CL}$. Further, $C'$ is both an ancestor of $\Box ^*(\mathbf{S }\rightarrow 0)$ and an LRES descendant of *C*. As $C'$ is an LRES descendant of *C*, by Lemma 3 the disjunction $D_1$ contains some negative extension literal $\lnot x_2'$ s.t. $x_2'\in {\mathscr {X}}_{e_2}$. Thus, by Lemma 4$\pi$ also contains a clause $C''=\Box ^*(\lnot y_{2}\vee D_2)$ where $y_2\in {\mathscr {X}}_{e_2}\cap (\{x_{\varepsilon }\} \cup {\mathscr {X}}_{{\mathscr {C}}_{\pm }})$ and $D_2\in \mathscr {CL}$, further $C''$ is both an ancestor of $\Box ^*(\mathbf{S }\rightarrow 0)$ and an LRES descendant of $C'$. As $y_1\in {\mathscr {X}}_{{\mathscr {C}}\pm }\cup \{x_{\varepsilon }\}$ it cannot appear positively in any literal clause. Hence, as $C''$ is an LRES descendant of $C'$ the disjunction $D_2$ must contain $\lnot y_{1}$. As $\Box ^*(\mathbf{S }\rightarrow 0)$ is a descendant of $C''$ both $\lnot y_1$ and $\lnot y_2$ must be resolved on at some stage in $\pi$. We proceed to show by induction on $|e_1|$ that this leads to a contradiction.

Suppose $|e_1|=1$, then $e_1=x_{\varepsilon }$ and $|e_2|>1$. The only initial clause containing a positive instance of $y_1=x_{\varepsilon }$ (resp. $y_2$) is $C_1'=\Box ^*(\mathbf{S }\rightarrow x_{\varepsilon })$ (resp. $C_2'=\Box ^*(y_2'\rightarrow \circ _a y_2)$). Further, no descendant of $C_1'$ (resp. $C_2'$) contains the positive literal $x_{\varepsilon }$ (resp. $y_2$). Hence, $\lnot x_{\varepsilon }$ must be resolved on using IRES1 and $\lnot y_2$ must be resolved on using either GEN1 or GEN3. As $\pi$ is in 1-start form $\lnot y_2$ must be resolved on first. Thus, either GEN1 or GEN3 must be applied to some set of clauses ${\mathscr {C}}'\supseteq \{C_2', C'''\}$ where $C'''$ is either $C''$ or some descendant of $C''$ containing $\lnot x_{\varepsilon }$ and $\lnot y_2$. However, the inference rules GEN1 and GEN3 both require every literal in $C'''$ to be resolved on simultaneously and so $\lnot x_{\varepsilon }$ must also be resolved on at this step in the refutation which is clearly impossible.

Now suppose $|e_1|>1$. For each $i\in [2]$, the only clause in ${\mathscr {C}}$ in which $\lnot y_i$ appears positively is $C_i'=\Box ^*( y_i'\rightarrow \circ _{a_i} y_i)$. Further, no descendant of $C_i'$ may contain a positive instance of $y_i$. As both $y_1$ and $y_2$ only appear positively in modal clauses they must be resolved on simultaneously by applying either GEN1 or GEN3 to some set ${\mathscr {C}}'\supseteq \{C_1',C_2',C'''\}$, where $C'''$ is either $C''$ or some descendant of $C''$ containing both $\lnot y_1$ and $\lnot y_2$. It follows that $a_1=a_2$ and at most one $C_1'$ and $C_2'$ is a negative modal clause as otherwise neither GEN1 nor GEN3 can be applied to ${\mathscr {C}}'$. In particular, we assume w.l.o.g. that $C_1'=\Box ^*(y_1'\rightarrow \Box _{a_1}y_1)$. Let $e_1'$ and $e_2'$ be the modal contexts of $y_1'$ and $y_2'$, respectively. As $C_1'$ and $C_2'$ are both initial clauses it follows from the definition of $\tau _0$ that $e_1=e_1'a$ and $e_2$ is either equal to $e_2'a$ or $e_1'(a_2,y_2)$. Hence, as $a_1=a_2$ we have $\sigma (a_1,a_2)=\sigma (a_1,(y_2,a_2))=a_1$ and so as $\sigma (e_1,e_2)$ is undefined $e_1'$ and $e_2'$ must be non-unifiable. Any clause inferred by applying either GEN1 or GEN3 to ${\mathscr {C}}'$ is a literal clause of the form $\Box ^*(\lnot y_1'\vee \lnot y_2'\vee D')$, where $D'\in \mathscr {CL}$. As $|e_1'|<|e_1|$ and $\sigma (e_1',e_2')$ is undefined it follows by induction that $\Box ^*(\mathbf{S }\rightarrow 0)$ is not a descendant of $C''$ and therefore cannot be a descendant of *C*. $\square$

#### Theorem 4

$\mathbf{K }_n$-Res $\equiv _p\,\mathbf{K }_{ml}$-Res $\equiv _p\,\mathbf{K }_{mc}$-Res.

#### Proof

Let $\phi$ be a **K**$_n$ formula in NNF. Translating $\phi$ into SNF, $\hbox {SNF}_{{ml}}$ and $\hbox {SNF}_{{mc}}$ we obtain three sets of clauses, denoted by ${\mathscr {C}}$, ${\mathscr {C}}_{ml}$ and ${\mathscr {C}}_{mc}$, respectively. There is a one to one correspondence between the clauses in each set. That is, for any $e\in {\mathscr {E}}_{{\mathscr {C}}}^*$ s.t. $|e|=m$:

$$\begin{aligned} \begin{array}{ccccc} (e: D)\in {\mathscr {C}}_{mc}&{}\iff &{}(m : D)\in {\mathscr {C}}_{ml}&{}\iff &{} \Box ^*( D)\in {\mathscr {C}},\\ (e: x\rightarrow \circ _a l)\in {\mathscr {C}}_{mc}&{}\iff &{} (m : x \rightarrow \circ _a l)\in {\mathscr {C}}_{ml}&{}\iff &{}\Box ^*( x\rightarrow \circ _a l)\in {\mathscr {C}},\\ (x_{\varepsilon }: x_{\varepsilon })\in {\mathscr {C}}_{mc}&{}\iff &{} (0 : x_{\varepsilon })\in {\mathscr {C}}_{ml}&{}\iff &{}\Box ^*(\mathbf{S }\rightarrow x_{\varepsilon })\in {\mathscr {C}}, \end{array} \end{aligned}$$

where $$x_{\varepsilon },x\in {\mathscr {X}}_{{\mathscr {C}}}$$, $$D\in \mathscr {CL}$$ and $$l\in {\mathscr {L}}$$.

($\ge _p$): Let $\pi _{mc}$ be a $\mathbf{K }_{mc}$-Res refutation of ${\mathscr {C}}_{mc}$. If we take $\pi _{ml}$ and $\pi$ to be the corresponding sequences of $\text {SNF}_{ml}$ and $\text {SNF}$ clauses, respectively, then we obtain a $\mathbf{K }_{ml}$-Res refutation of ${\mathscr {C}}_{ml}$ and a $\mathbf{K }_n$-Res refutation of ${\mathscr {C}}$, respectively.

($\le _p$): Now suppose $\pi$ is a $\mathbf{K }_n$-Res refutation of ${\mathscr {C}}$ in 1-start form. Let $\pi '=C_1,\dots ,C_m$ be the sequence of clauses obtained by deleting every clause with non-unifiable modal contexts from $\pi$. By Lemma 5$\pi '$ is a $\mathbf{K }_n$-Res refutation of ${\mathscr {C}}$. To prove that the analogous sequence of $\hbox {SNF}_{{mc}}$ clauses^5^ is a $\mathbf{K }_{mc}$-Res refutation of ${\mathscr {C}}_{mc}$ we must verify that each clause in $\pi '$ is inferred from a set of clauses whose modal contexts agree with those required to apply the corresponding inference rule of ${\mathbf {K}}_{mc}$-Res.

Note that as $\pi '$ is in 1-start form it cannot contain any clauses inferred using IRES2. Suppose some *C* in $\pi '$ is inferred from two clauses $C_1$ and $C_2$ using IRES1. Then, as $\pi '$ is in 1-start form we can assume w.l.o.g. that $C_{1}=\Box ^*(\lnot x_{\varepsilon })$ and $C_2=\Box ^*({\mathbf {S}}\rightarrow x_{\varepsilon })$. Clearly $C_1$ and $C_2$ both have modal context $x_{\varepsilon }$ and so LRES can be applied to the analogous $\hbox {SNF}_{{mc}}$ clauses to infer $(x_{\varepsilon }:0)$.

Suppose $C=\Box ^*C'$ is inferred by applying LRES to some $C_1$ and $C_2$ in $\pi '$. Let $\{e_1,\dots ,e_{n_1}\}$ and $\{e_{1}',\dots , e_{n_2}'\}$ be the sets of all modal contexts of $C_1$ and all modal contexts of $C_2$, respectively. Then, by Lemma 3 the clause *C* must contain negative extension variables with modal contexts $e_1,\dots ,e_{n_1},e_{1}',\dots , e_{n_2}'$. As *C* is unifiable there exists some $e\in {\mathscr {E}}_{{\mathscr {C}}}^*$ s.t. $e=\sigma (e_1,\dots ,e_{n_1},e_{1}',\dots , e_{n_2}')$. Hence, we can apply LRES to the analogous $\hbox {SNF}_{{mc}}$ clauses to infer (*e* : *C*).

Suppose $C=\Box ^*C'$ is inferred by applying MRES (resp. GEN2) to some $C_1$ and $C_2$ (resp. $C_1$, $C_2$ and $C_3$). As $C_1$ and $C_2$ (resp. $C_1$, $C_2$ and $C_3$) are modal clauses they must each have a single modal context. Let $e_1$ and $e_2$ (resp. $e_1$, $e_2$ and $e_3$) be the modal contexts of $C_1$ and $C_2$ (resp. $C_1$, $C_2$ and $C_3$), respectively. It follows from the definition of MRES (resp. GEN2) that *C* has modal contexts $e_1$ and $e_2$ (resp. $e_1$, $e_2$ and $e_3$). Further, as *C* has unifiable contexts $\sigma (e_1,e_2)$ (resp. $\sigma (e_1,e_2,e_3)$) is defined. Hence, we can apply MRES (resp. GEN2) to $C_1$ and $C_2$ (resp. $C_1$, $C_2$ and $C_3$) to infer $(\sigma (e_1,e_2): C)$ (resp. $(\sigma (e_1,e_2,e_3): C)$).

Finally suppose $C=\Box ^*(\lnot l_1'\vee \dots \vee \lnot l_{z+1}')$ is inferred using GEN1 (resp. GEN3). Then, *C* is inferred from *z* positive modal clauses $C_1=\Box ^*(l_1'\rightarrow \Box _a l_1),\dots ,C_z=\Box ^*(l_z'\rightarrow \Box _a l_z)$, one negative modal clause $C_{z+1}=\Box ^*(l_{z+1}'\rightarrow \Diamond _a l_{z+1})$ and one literal clause $C_{z+2}=\Box ^*(\lnot l_1\vee \dots \vee \lnot l_{z+1})$ (resp. $C_{z+2}=\Box ^*(\lnot l_1\vee \dots \vee \lnot l_{z})$). Each of the modal clauses must have a single modal context, hence we let $e_1,\dots ,e_{z+1}$ be the modal contexts of $C_1,\dots ,C_{z+1}$, respectively. By the definition of GEN1 (resp. GEN3) *C* has modal contexts $e_1,\dots , e_{z+1}$ and so as *C* has unifiable modal contexts there exists some $e\in {\mathscr {E}}_{{\mathscr {C}}}^*$ s.t. $\sigma (e_{1},\dots ,e_{z+1})=e$. Further, it follows from the definition of $\tau _0$ that the set of modal contexts of $C_{z+2}$ is a subset of $\{e_1a,\dots ,e_{z}a,e_{z+1}(l_{z+1},a)\}$ (resp. $\{e_{1}a,\dots ,e_{z}a\}$). Hence, we can apply GEN1 (resp. GEN3) to the set of $\hbox {SNF}_{{mc}}$ clauses corresponding to $\{C_1,\dots ,C_{z+2}\}$ to infer (*e* : *C*).

$$\square $$

## Game Theoretic Lower Bound Technique

In this section we introduce an asymmetric two-player game based on those of [18, 19, 57]. This game is played by a Prover and a Delayer, on an unsatisfiable set of $\hbox {SNF}_{{mc}}$ clauses ${\mathscr {C}}$. Prover’s goal is to construct a countermodel for a certain set of clauses ${\mathscr {D}}\subseteq \{C\mid {\mathscr {C}}\vdash _{{\mathbf {K}}_{mc}\text {-Res}}C\}$. The set ${\mathscr {D}}$ is defined in such a way as to ensure that it is unsatisfiable iff ${\mathscr {C}}$ is, and so it will always be possible for Prover to construct such a model. Hence, Delayer’s goal is not to prevent Prover from doing so, but to score as many points as possible before the game ends. We further show that lower bounds on the proof size required to refute some unsatisfiable set of $\hbox {SNF}_{{mc}}$ clauses can be obtained indirectly by showing a lower bound on Delayer’s score. In particular, such lower bounds are lower bounds on the number of modal proof steps required to refute ${\mathscr {C}}$.

Before formally defining our two-player game we must extend the set of words we use to specify the modal contexts of a given set of $\hbox {SNF}_{{mc}}$ clauses ${\mathscr {C}}$. This is because we need to be able to specify the modal context of every literal *l* that appears in a clause of the form $(e: x\rightarrow \Diamond _a l)$. Clearly if $l\in {\mathscr {X}}_{{\mathscr {C}}}$ then we can do this using the set of words ${\mathscr {E}}_{{\mathscr {C}}}^*$ (*l* has modal context *e*(*a*, *l*)), however if $l\not \in {\mathscr {X}}_{{\mathscr {C}}}$ then its modal context cannot be described by any word in ${\mathscr {E}}_{{\mathscr {C}}}^*$. Hence, we have the following definition.

### Definition 20

Let ${\mathscr {C}}$ be a set of $\hbox {SNF}_{{mc}}$ clauses. We define:

$$\begin{aligned}&{\mathscr {L}}_{{\mathscr {C}}-}=\{(x',x)\in {\mathscr {L}}\times {\mathscr {L}} \mid (e: x'\rightarrow \Diamond _a x)\in {\mathscr {C}}\},\\&\quad \bar{{\mathscr {E}}}_{{\mathscr {C}}}={\mathscr {E}}_{{\mathscr {C}}} \cup ({\mathscr {A}}\times {\mathscr {L}}_{{\mathscr {C}}-}). \end{aligned}$$

We say each element of $\bar{{\mathscr {E}}}_{{\mathscr {C}}}$ is a *context marker* for ${\mathscr {C}}$.

Now if $(e: x\rightarrow \Diamond _a l)\in {\mathscr {C}}$ then clearly $(x,l)\in {\mathscr {L}}_{{\mathscr {C}}-}$ and so the modal context of *l* is given by the word *e*(*a*, (*x*, *l*)). Therefore in this section we use the set of finite words over $\bar{{\mathscr {E}}}_{{\mathscr {C}}}$ to specify the modal contexts of clauses and variables.

We further extend the definition of $\sigma$ so that $\sigma :\bar{{\mathscr {E}}}_{{\mathscr {C}}}^*\times \dots \times \bar{{\mathscr {E}}}_{{\mathscr {C}}}^*\mapsto \bar{{\mathscr {E}}}_{{\mathscr {C}}}^*$ and for $c_1,\dots ,c_n\in \bar{{\mathscr {E}}}_{{\mathscr {C}}}$ we have $\sigma (c_1,\dots , c_n)=(a,(x',x))$ if for some $j\in [n]$ we have $y_j=(a,(x',x))$ and for all $k\ne j$ we have $c_k=a$ or $c_k=(a,(x',x))\}$. We also extend the definition of the reachability of a world (Definition 11) to $\bar{{\mathscr {E}}}_{{\mathscr {C}}}^*$ in the obvious way^6^.

The following four definitions give us some convenient notation.

### Definition 21

Let $\Sigma$ be a set and let $w\in \Sigma ^*$. We say *w* is a *prefix* of some word $u\in \Sigma ^*$ (denoted $w\sqsubseteq u$) if and only if $u=wv$ where $v\in \Sigma ^*$. We say *w* is a *proper prefix* of some word $u\in \Sigma ^*$ (denoted $w\sqsubset u$) if and only if *w* is a prefix of *u* and $w\ne u$.

We say *w* is a *suffix* of some word $u\in \Sigma ^*$ (denoted $w\sqsupseteq u$) if $w=uv$ where $v\in \Sigma ^*$. We say *w* is a *proper suffix* of some word $u\in \Sigma ^*$ (denoted $w\sqsupset u$) if *w* is a suffix of *u* and $w\ne u$.

We say *u* is a *subword* of *w* if $w=w_1uw_2$ for some $w_1,w_2\in \Sigma ^*$, denoted $u\vartriangleleft w$.

### Definition 22

Let ${\mathscr {C}}$ be a set of $\hbox {SNF}_{{mc}}$ clauses and let $e\in \bar{{\mathscr {E}}}_{{\mathscr {C}}}^*$. We define:

$$\begin{aligned} \bar{{\mathscr {E}}}_{e\sqsubset } =\{e'\in {\mathscr {E}}_{{\mathscr {C}}}^*\mid \sigma (e,e'')\in \bar{{\mathscr {E}}}_{{\mathscr {C}}}^* \text { and }e''\sqsubset e'\}. \end{aligned}$$

The sets $\bar{{\mathscr {E}}}_{e\sqsupset }$, , , ,  and $\bar{{\mathscr {E}}}_{e=}$ are defined similarly.

### Definition 23

Let ${\mathscr {C}}$ be a set of $\hbox {SNF}_{{mc}}$ clauses. For each $e\in \bar{{\mathscr {E}}}_{{\mathscr {C}}}^*$ we define:

$$\begin{aligned} L_e&=\{(e': C)\in {\mathscr {C}}\mid C\in \mathscr {CL} \text { and } \sigma (e,e')\in \bar{{\mathscr {E}}}_{{\mathscr {C}}}^*\},\\ {\mathscr {C}}_{e}&=L_e\cup \{(e': x'\rightarrow \circ _a x) \in {\mathscr {C}}\mid \sigma (e,e'')\in \bar{{\mathscr {E}}}_{{\mathscr {C}}}^* \text { where }e'' \text { is the modal context of } x\},\\ N_{e}&=\{(e': x'\rightarrow \Diamond _a x)\in {\mathscr {C}}\mid \sigma (e,e')\in \bar{{\mathscr {E}}}_{{\mathscr {C}}}^*\}. \end{aligned}$$

Then, the set $L_e$ consists of all literal clauses whose modal context is unifiable with *e* and the set $N_e$ is the set of all negative modal clauses whose modal context is unifiable with *e*. The set ${\mathscr {C}}_{e}$ is the set of all clauses to which a rule of $\mathbf{K }_{mc}$-Res can be applied to resolve on some variable with modal context *e* (not to be confused with the set of all clauses whose modal context is unifiable with *e*).

### Query Sets

Several different Prover–Delayer games have been used to prove lower bounds for tree-like propositional resolution (cf. [18, 19, 57]). Such games are played over an unsatisfiable propositional formula $\phi$ in CNF. Over the course of a game on $\phi$ Prover and Delayer build a propositional countermodel for $\phi$ (that is, a partial assignment $\alpha$ to the variables in $\phi$ s.t. for some propositional clause $C\in \phi$ we have $\alpha (C)=0$). At each round Prover queries some as yet unassigned variable in $\phi$ and $\alpha$ is extended to include an assignment for this variable. The game ends when $\alpha (C)=0$ for some propositional clause *C* in $\phi$.

Similarly over the course of a modal game (as defined in Sect. 6.2) played on an unsatisfiable set of $\hbox {SNF}_{{mc}}$ clauses ${\mathscr {C}}$ Prover and Delayer build a pointed countermodel $\langle M,w_{\varepsilon }\rangle$ for some set of clauses ${\mathscr {D}}\subseteq {\mathscr {C}}\cup \{C\mid {\mathscr {C}}\vdash _{{\mathbf {K}}_{mc}\text {-Res}} C\}$. The exact definition of ${\mathscr {D}}$ is given in Sect. 6.2, however for now it suffices to note that ${\mathscr {D}}$ is unsatisfiable iff ${\mathscr {C}}$ is unsatisfiable. At the start of any such game Prover and Delayer have a model consisting of a single world $w_{\varepsilon }$. New worlds are added to this model at each round of the game. Hence, the key difference between the previously proposed propositional games and our modal game is that at each round Prover queries a world in the current model, instead of a variable in ${\mathscr {C}}$. Querying a world *w* essentially means asking whether or not to add a new world $w'$ which is accessible from *w* to the model and if so for which context marker $b\in \bar{{\mathscr {E}}}_{{\mathscr {C}}}$ is $w'$ *b*-accessible from *w*. If at a given round no world is added to the model then the game ends.

Now, suppose some pointed model $\langle M,w_{\varepsilon }\rangle$ is a countermodel for a set of $\hbox {SNF}_{{mc}}$ clauses ${\mathscr {C}}$. Then, clearly there must exists some $C\in {\mathscr {C}}$ for which $(M,w_{\varepsilon })\not \models C$. If *C* is a negative modal clause then $C=(e: l\rightarrow \Diamond _a l')$ for some $e\in \bar{{\mathscr {E}}}_{{\mathscr {C}}}^*$, $l,l'\in {\mathscr {L}}$ (resp. $l\in {\mathscr {L}}$, $l'\in {\mathscr {X}}_{{\mathscr {C}}}$) and $a\in {\mathscr {A}}$. Hence, as $(M,w_{\varepsilon })\not \models C$, the model *M* must contain a world *w* which is *e*-accessible from $w_{\varepsilon }$ and for which $V(w)(l)=1$, but no world $w'$ that is $(a,(l,l'))$-accessible (resp. $(a,l')$-accessible) from *w* and so we say that $\langle M,w_{\varepsilon }\rangle$ *modally falsifies* *C*. Otherwise *C* is either a positive modal clause or a literal clause. In either case *M* must fail to satisfy *C* because of its valuation functions^7^ and so we say that $\langle M,w_{\varepsilon }\rangle$ *propositionally falsifies* *C*.

Obviously, if a model $\langle M,w_{\varepsilon }\rangle$ modally falsifies some clause $C=(e:l\rightarrow \Diamond _a l')$, where $e\in \bar{{\mathscr {E}}}_{{\mathscr {C}}}^*$, $l\in {\mathscr {X}}_{{\mathscr {C}}}$ and $l'\in {\mathscr {L}}{\setminus }{\mathscr {X}}_{{\mathscr {C}}}$ (resp. $l'\in {\mathscr {X}}_{{\mathscr {C}}}$) then we can obtain a new model which satisfies *C* by adding a new world $w'$ which is $e(a,(l,l'))$-accessible (resp. $e(a,l')$-accessible) from $w_{\varepsilon }$. Whereas if $\langle M,w_{\varepsilon }\rangle$ propositionally falsifies some clause *C* then no extension of *M* can possibly satisfy *C*. Given this it is natural to require that the countermodel for ${\mathscr {D}}\subseteq {\mathscr {C}}\cup \{C\mid {\mathscr {C}}\vdash _{{\mathbf {K}}_{mc}\text {-Res}} C\}$ built over the course of a modal game on ${\mathscr {C}}$ propositionally falsifies some clause $C\in {\mathscr {D}}$.

Recall that our modal game ends at a round where some world *w* is queried only if Prover chooses not to add a new world to the model. Hence, we add the condition that after querying a world *w* Prover may only choose not to add a world to the model $\langle M,w_{\varepsilon }\rangle$ if this model already propositionally falsifies some $C\in {\mathscr {D}}$. We shall see in Sect. 6.2 that the exact definition of ${\mathscr {D}}$ depends on the worlds queried in the previous rounds of the game. Furthermore, ${\mathscr {D}}$ is defined so that every negative modal clause in ${\mathscr {D}}$ is satisfied by $\langle M,w_{\varepsilon }\rangle$ and so ${\mathscr {D}}$ is propositionally falsified by $\langle M,w_{\varepsilon }\rangle$ whenever $(M,w_{\varepsilon })\not \models {\mathscr {D}}$.

Finally, to ensure that the game always terminates we require that every new world added to the model is *b*-accessible, for some $b\in \bar{{\mathscr {E}}}_{{\mathscr {C}}}{\setminus }{\mathscr {A}}$. Note that this ensures that each new world corresponds to some negative modal clause in ${\mathscr {C}}$, preventing Prover and Delayer from adding new worlds to the model which tell us nothing about the satisfiability of ${\mathscr {C}}$.

We formalise these restrictions by requiring that whenever Prover queries a world *w* that is *e*-accessible from the root world $w_{\varepsilon }$, she must also query some *query set* for *e* w.r.t. ${\mathscr {D}}$ (Definition 24). A query set is a set of context markers. We allow Prover to choose not to add any world to the model at a given round only if she has queried the set $Q_e=\emptyset$. Otherwise, Prover must add a world $w'$ that is *b*-accessible from *w* for some $b\in Q_{e}$ to the model.

#### Definition 24

Let ${\mathscr {C}}$ be an unsatisfiable set of $\hbox {SNF}_{{mc}}$ clauses and let $e\in (\bar{{\mathscr {E}}}_{{\mathscr {C}}}{\setminus }{\mathscr {A}})^*$. We say that a set $Q_{e}$ is a *query set* for *e* w.r.t. ${\mathscr {C}}$ if and only if it satisfies the following constraints:

- $$ Q_{e}\subseteq \{(a,(x',x))\in {\mathscr {A}}\times {\mathscr {L}}_{{\mathscr {C}}-}\mid (e':x'\rightarrow \Diamond _a x)\in N_e\}\cup \{(a,x)\in {\mathscr {A}}\times {\mathscr {X}}_{{\mathscr {C}}-}\mid (e':x''\rightarrow \Diamond _a x)\in N_e\}. $$
- For every model $M=(W,R_{a_1},\dots , R_{a_n}, V)$ and every world $w\in W$ either *M* contains no world that is *e*-accessible from *w* or:
  $$\begin{aligned} (M,w)\not \models \bigcup _{e_1\in \bar{{\mathscr {E}}}_{e\sqsupseteq }}{\mathscr {C}}_{e_1}\cup \bigcup _{b\in Q_e}{\mathscr {M}}_{eb}, \end{aligned}$$
  where for each $b\in Q_{e}$ we define ${\mathscr {M}}_{eb}$ to be the set of all clauses that can be inferred by applying some modal rule to some set of clauses $\mathscr {C'}$ s.t. for every $C\in {\mathscr {C}}'$ either $C\in {\mathscr {C}}_{eb}$ or $\bigcup _{e_1\in \bar{{\mathscr {E}}}_{eb\sqsubseteq }}{\mathscr {C}}_{e_1} \vdash _{{\mathbf {K}}_{mc}\text {-Res}} C$.

Consider the unsatisfiable formula $\Diamond _a(x\wedge \lnot x)\wedge (\Diamond _a y\vee \Diamond _a z)$. The corresponding set of $\hbox {SNF}_{{mc}}$ clauses is:

$$\begin{aligned} {\mathscr {C}}&=\{(\varepsilon : x_{\varepsilon }), \;(\varepsilon : x_{\varepsilon }\rightarrow \Diamond _a x_1),\; ((a,x_1): \lnot x_1\vee x),\; ((a,x_1): \lnot x_1\vee \lnot x),\\&\qquad (\varepsilon : \lnot x_{\varepsilon }\vee x_2\vee x_3),\;(\varepsilon : x_2\rightarrow \Diamond _a y),\;(\varepsilon : x_3\rightarrow \Diamond _a z)\}. \end{aligned}$$

It is not hard to see that every unsatisfiable subset of ${\mathscr {C}}$ must be a superset of ${\mathscr {C}}_{(a,x_1)}$. Hence, $\{(a,x_1)\}$, $\{(a,x_1), (a,(x_2,y))\}$, $\{(a,x_1), (a,(x_2,y)), (a,(x_3,z))\}$, and $\{(a,x_1),(a,(x_3,z))\}$ are all query sets for $\varepsilon$ w.r.t. ${\mathscr {C}}$.

Further, any model that satisfies ${\mathscr {C}}_{\varepsilon }\cup N_{\varepsilon }=\{(\varepsilon : x_{\varepsilon }),\;(\varepsilon : x_{\varepsilon }\rightarrow \Diamond _a x_1),\;(\varepsilon : \lnot x_{\varepsilon }\vee x_2\vee x_3),\;(\varepsilon : x_2\rightarrow \Diamond _a y),\;(\varepsilon : x_3\rightarrow \Diamond _a z)\}$ at some world $w_{\varepsilon }$ must also contain a world *w* which is $(a,x_1)$-accessible from $w_{\varepsilon }$. Hence, as every query set, $Q_{\varepsilon }$ for $\varepsilon$ w.r.t. ${\mathscr {C}}$ contains $(a,x_1)$ the following statement holds: “every model that satisfies ${\mathscr {C}}_{\varepsilon }\cup N_{\varepsilon }$ must contain a world that is *b*-accessible from for some $b\in Q_{\varepsilon }$”. We will see in the following proposition that an analogous statement holds for any modal context *e* and any set of clauses ${\mathscr {C}}$. Hence, we can think of a query set for *e* w.r.t. ${\mathscr {C}}$ as representing a set of worlds $W'$ such that any model *M* that could possibly satisfy ${\mathscr {C}}$ contains some $w\in W'$.

#### Proposition 2

Let ${\mathscr {C}}$ be an unsatisfiable set of $\hbox {SNF}_{{mc}}$ clauses and let $Q_e$ be a query set for some modal context $e\in \left( \bar{{\mathscr {E}}}_{{\mathscr {C}}}{\setminus }{\mathscr {A}} \right) ^*$ w.r.t. ${\mathscr {C}}$. Further, let $M=(W,R_{a_1},\dots , R_{a_n}, V)$ and $w_{\varepsilon }\in W$. If *W* contains some world $w_1$ that is *e*-accessible from $w_{\varepsilon }$ and:then there exists some $w_2\in W$ that is *eb*-accessible from $w_{\varepsilon }$, for some $b\in Q_e$.

#### Proof

As $Q_e$ is a query set for *e* and $w_1\in W$ is *e*-accessible from $w_{\varepsilon }$ by part (b) of Definition 24 we have $(M,w_{\varepsilon })\not \models \bigcup _{e_1\in \bar{{\mathscr {E}}}_{e\sqsupseteq }}{\mathscr {C}}_{e_1}\cup \bigcup _{b\in Q_{e}} {\mathscr {M}}_{eb}$. Hence, as by assumption  and  there must exist some $C\in {\mathscr {M}}_{eb}$ s.t. $(M,w_{\varepsilon })\not \models C$, where $b\in Q_e$. As any such clause is inferred by applying some modal rule of $\mathbf{K }_{mc}$-Res to some set of clauses whose modal contexts are unifiable with *eb*, *C* must be of the form $(e':x_1\vee \dots \vee \lnot x_z\vee \lnot y')$ where $e'\in \bar{{\mathscr {E}}}_{e=}$ and $y'\in {\mathscr {X}}_{{\mathscr {C}}}$ s.t. $(e'':y'\rightarrow \Diamond _a y'')\in N_e$ and either $b=(a,(y',y''))$ or $b=(a,y'')$. And so there must exist some $w\in W$ s.t. $V(w)(y')=1$ and *w* is $e'$-accessible from $w_{\varepsilon }$. Further, as $(M,w)\models N_e$ we have $(M,w_{\varepsilon })\models (e'':y'\rightarrow \Diamond _a y'')$ and so $V(w)(\Diamond _a y'')=1$. That is, there exists some $w_2\in W$ s.t. $V(w_2)(y'')=1$ and $(w,w')\in R_a$ and so $w_2$ is *b*-accessible from *w*.

To prove that $w_2$ is *eb*-accessible from $w_{\varepsilon }$ we show by contradiction that *w* is *e*-accessible from $w_{\varepsilon }$. Suppose that *w* is not *e*-accessible from $w_{\varepsilon }$, then clearly $e'\ne e$. By Remark 2 we have $\bigcup _{e_1\in \bar{{\mathscr {E}}}_{e'y\sqsubseteq }}{\mathscr {C}}_{e_1} \vdash _{{\mathbf {K}}_{mc}\text {-Res}} C$ and so by the soundness of $\mathbf{K }_{mc}$-Res $(M,w_{\varepsilon })\not \models \bigcup _{e_1\in \bar{{\mathscr {E}}}_{e'b\sqsubseteq }}{\mathscr {C}}_{e_1}$. Clearly  and so , contradicting our original assumption. $\square$

### Prover–Delayer Game

In this section, we define our two-player game which is played by a Prover (who, for clarity is female) and a Delayer (male) on some unsatisfiable set of $\hbox {SNF}_{{mc}}$ clauses ${\mathscr {C}}$. Recall that Prover’s goal is to construct a countermodel for a given set of clauses ${\mathscr {D}}\subseteq \{C\mid {\mathscr {C}}\vdash _{{\mathbf {K}}_{mc}\text {-Res}}C\}$. Further, this model must propositionally falsify some $C\in {\mathscr {D}}$. The set of clauses ${\mathscr {D}}$ that Prover is trying to build a countermodel for depends on the modal context of the game and so changes throughout the game.

At the beginning of the game, we have a pointed model consisting of a single world with modal context $\varepsilon$ and the set of clauses ${\mathscr {C}}$. Further, Delayer’s score is 0 and the modal context of the game is $\varepsilon$. At each round, if the game has modal context *e* and we have the set of clauses ${\mathscr {D}}$ then Prover chooses some query set $Q_e$ for *e* w.r.t. ${\mathscr {D}}$. If $Q_e=\emptyset$ then the game ends and Prover wins. Otherwise the round continues with Prover adding a new world with modal context *ec* to the model, where $c\in Q_e$. Before Prover adds a new world to the model Delayer gives a weight to each $c\in Q_e$. The lower the weight Delayer gives to a particular $c\in Q_e$ the more points he will score if Prover chooses to add a world with modal context *ec*. At the end of the round, Delayer’s score and the set of clauses are updated, and the modal context of the game is changed to *ec*.

Formally, a game on some unsatisfiable set of $\hbox {SNF}_{{mc}}$ clauses ${\mathscr {C}}$ is played as follows. At the start of the game there exists a pointed model, $\langle M^1,w_{\varepsilon }\rangle$ where $M^1=(W^1,R_{a_1}^1,\dots , R_{a_n}^1,V^1)$, $W^1=\{w_{\varepsilon }\}$, $R_{a_i}^1=\emptyset$ for all $i\in [n]$ and $V^1(w_{\varepsilon })(x_{\varepsilon })=1$. Further, we have modal context $e^1=\varepsilon$, the set ${\mathscr {D}}^1={\mathscr {C}}$ and Delayers score is $s^1=0$. The *i*th round of the game is played as follows:

- Prover fixes some query set $Q_{e^{i}}$ for $e^{i}$ w.r.t. ${\mathscr {D}}^i$.
- If $Q_{e^{i}}=\emptyset$ then the game ends.
- Otherwise Delayer assigns a weight $p_{c}$ to each $c\in Q_{e^{i}}$ so that $\sum _{c\in Q_{e^{i}}}p_{c}=1$.
- Prover picks some $c=(a',z)\in Q_{e^{i}}$ and the status of the game is updated as follows:
  $$\begin{aligned}&e^{i+1}= e^{i}c, \;\;\;s^{i+1}=s^{i}+\log \left( \frac{1}{p_c}\right) ,\; \mathscr {D}^{i+1}=\bigcup _{e\in \bar{\mathscr {E}}_{e^i\sqsupseteq }} \mathscr {D}^{i}_{e}\cup \bigcup _{e\in \bar{\mathscr {E}}_{e^{i+1} \sqsubseteq }}\mathscr {C}_{e}\cup \bigcup _{b\in Q_{e^i}{\setminus }\{c\}} \mathscr {M}_{e^ib},\\&W^{i+1}= W^{i}\cup \{w_{e^{i}c}\},\;\;\;\; R_a^{i+1}={\left\{ \begin{array}{ll} R_{a}^{i}\cup \{(w_{e^{i}},w_{e^{i}c})\} &{} \text {if } a=a_c,\\ R_a^{i} &{}\text {otherwise,} \end{array}\right. }\\&V^{i+1}(w_{e^{i}c})(x)= 1 \text { if either } z=x \text { or } z=(x',x). \end{aligned}$$
  Where for each $b\in Q_{e^i}$ the set ${\mathscr {M}}_{e^ib}$ is defined as in part (b) Definition 24.

Note that our game can only be played if at each round the modal context $e^i$ and the set of clauses ${\mathscr {D}}^i$ are s.t. there exists a query set for $e^i$ w.r.t. ${\mathscr {D}}^i$. We will see in Proposition 3 that this is always the case.

At each round of the game Delayer claims that the subset of ${\mathscr {D}}^i$ consisting of every clause whose modal context is a prefix of $e^i$ is satisfied by $\langle M^i,w_{\varepsilon }\rangle$, and that some extension of $M^i$ satisfies ${\mathscr {D}}^i$. Prover then picks some query set $Q_{e^i}$ for $e^i$ w.r.t. ${\mathscr {D}}^i$ and proceeds in one of two ways. If $Q_{e^i}=\emptyset$ then by definition no model containing a world *w* which is $e^i$-accessible from $w_{\varepsilon }$ satisfies . As $M^i$ contains such a world no extension of $M^i$ can possibly satisfy ${\mathscr {D}}^i$ and so Prover sees that Delayer must be lying and ends the game. Note that every negative modal clause in $\bigcup _{e\in \bar{{\mathscr {E}}}_{e^i\sqsupseteq }}{\mathscr {C}}_{e}$ is satisfied by $\langle M^i, w_{\varepsilon }\rangle$ so $M^i$ must propositionally falsify .

If $Q_{e^i}\ne \emptyset$ then Prover first notes that $M^i$ contains a world $w_{e^i}$ which is $e^i$-accessible from $w_{\varepsilon }$. Hence, by Proposition 2 any extension of $M^i$ can only satisfy the set of negative clauses with modal context $e^i$, i.e. $N_{e^i}\subseteq {\mathscr {D}}^i$ at $w_{\varepsilon }$ if it contains a world that is $e^ib$-accessible from $w_{\varepsilon }$ for some $b\in Q_{e^i}$. Hence, $\langle M^i, w_{\varepsilon } \rangle$ is not a model for ${\mathscr {D}}^i$ and so Prover adds some such world to $M^i$ to create a new model $M^{i+1}$, which could potentially satisfy $N_{e^i}$, and so ${\mathscr {D}}^i$.

In Proposition 3, we prove that any countermodel for a set ${\mathscr {D}}^i$ is also a countermodel for ${\mathscr {C}}$. Hence, the model $M^k$ built over the course of some game with exactly *k* rounds, and every model that extends $M^k$ are countermodels for ${\mathscr {C}}$. Note that it is not necessarily the case that no previously considered model $M^i$ where $i\in [k-1]$ was a countermodel for ${\mathscr {C}}$, as the rules of the game do not force Prover to set $Q_{e^i}=\emptyset$ whenever it is a valid query set for $e^i$. However, if Prover wishes to minimise Delayers score she would always choose to set $Q_{e^i}=\emptyset$ at the first opportunity as this ends the game without allowing Delayer to score any more points.

The following proposition ensures that the game can actually always be played.

#### Proposition 3

Let ${\mathscr {C}}$ be a set of unsatisfiable clauses. If a game is played on ${\mathscr {C}}$ then:

- For each *i*, if $(M,w)\not \models {\mathscr {D}}^i$ then $(M,w)\not \models {\mathscr {C}}$.
- For each *i*, the set ${\mathscr {D}}^i$ is satisfiable iff ${\mathscr {C}}$ is satisfiable.
- There exists a query set for each ${\mathscr {C}}^i\cup \bigcup _{e\in \bar{{\mathscr {E}}}_{e^i\sqsubset }} {\mathscr {C}}_{e}$.

#### Proof

- As ${\mathscr {D}}^1={\mathscr {C}}$ it follows by definition that for each *i* every clause in ${\mathscr {D}}^i$ is either in ${\mathscr {C}}$ or is $\mathbf{K }_{mc}$-Res provable from ${\mathscr {C}}$. It is not hard to see that each of the rules of $\mathbf{K }_{mc}$-Res preserve satisfiability^8^ . Hence, for each *i*, if $(M,w)\not \models {\mathscr {D}}^i$ then $(M,w)\not \models {\mathscr {C}}$.
- This follows from parts (a) and (c).
- This can be seen by induction on *i*. If $i=1$ then ${\mathscr {D}}^i={\mathscr {C}}$. As ${\mathscr {C}}$ is unsatisfiable and $\mathbf{K }_{mc}$-Res is complete it follow that if we let:
  $$\begin{aligned} Q_{\varepsilon }=\,&\{(a,(x',x))\in {\mathscr {A}} \times {\mathscr {L}}_{{\mathscr {C}}-}\mid (\varepsilon :x'\rightarrow \Diamond _a x)\in N_{\varepsilon }\}\cup \\&\{(a,x)\in {\mathscr {A}}\times {\mathscr {X}}_{{\mathscr {C}}-}\mid (\varepsilon :x''\rightarrow \Diamond _a x)\in N_{\varepsilon }\}. \end{aligned}$$
  then ${\mathscr {C}}_{\varepsilon }\cup \bigcup _{b\in Q_{\varepsilon }}{\mathscr {M}}_{b}$ is unsatisfiable. Hence, $Q_{\varepsilon }$ is a query set for $\varepsilon$ w.r.t. ${\mathscr {D}}^1$.
  If $i>1$ then ${\mathscr {D}}^i=\bigcup _{e\in \bar{{\mathscr {E}}}_{e^{i-1}\sqsupseteq }} {\mathscr {D}}^{i-1}_{e}\cup \bigcup _{e\in \bar{{\mathscr {E}}}_{e^{i} \sqsubseteq }}{\mathscr {C}}_{e}\cup \bigcup _{b\in Q_{e^{i-1}}{\setminus }\{c\}}{\mathscr {M}}_{e^{i-1}b}$, where $Q_{e^i}$ is a query set for $e^{i-1}$ w.r.t. ${\mathscr {D}}^{i-1}$ and $c\in Q_{e^{i-1}}$ s.t. $e^i=e^{i-1}c$. That ${\mathscr {D}}^i$ is well defined follows from the inductive hypothesis. As $Q_{e^{i-1}}$ is a query set for $e^{i-1}$ the set $\bigcup _{e\in \bar{{\mathscr {E}}}_{e^{i-1}\sqsupseteq }}{\mathscr {D}}_e^{i-1}\cup \bigcup _{b\in Q_{e^{i-1}}}{\mathscr {M}}_{e^{i-1}b}$ must be unsatisfiable. Note that every clause in ${\mathscr {M}}_{e^{i-1}c}$ must be inferred from some set ${\mathscr {C}}'\subseteq {\mathscr {C}}_{e^i}\cup \bigcup _{b\in Q_{e^i}}{\mathscr {M}}_{e^ib}$, where:
  $$\begin{aligned} Q_{e^i}=\,&\{(a,(x',x))\in {\mathscr {A}}\times {\mathscr {L}}_{{\mathscr {C}}-}\mid (e':x'\rightarrow \Diamond _a x)\in N_{e^i}\}\cup \\&\{(a,x)\in {\mathscr {A}}\times {\mathscr {X}}_{{\mathscr {C}}-}\mid (e':x''\rightarrow \Diamond _a x)\in N_{e^i}\}. \end{aligned}$$
  It follows by the completeness of $\mathbf{K }_{mc}$-Res that $\bigcup _{e\in \bar{{\mathscr {E}}}_{e^{i-1}\sqsupseteq }}{\mathscr {D}}_e^{i-1} \cup \bigcup _{b\in Q_{e^{i-1}{\setminus }\{c\}}}{\mathscr {M}}_{e^{i-1}b} \cup {\mathscr {C}}_{e^i} \cup \bigcup _{b\in Q_{e^i}}{\mathscr {M}}_{e^ib}$ is unsatisfiable. Hence, $Q_{e^i}$ is a query set for $e^i$ w.r.t. ${\mathscr {D}}^i$.

$$\square $$

### Modal Decision Trees

To use our two-player game to obtain modal proof size lower bounds we need to establish a connection between it and the number of modal resolution steps required to refute a formula using $\mathbf{K }_{mc}$-Res. Hence, in this section we introduce *modal decision trees*. The number of vertices in a modal decision for some unsatisfiable set of $\hbox {SNF}_{{mc}}$ clauses ${\mathscr {C}}$ is connected to both the number of modal resolution steps required to refute ${\mathscr {C}}$ (Proposition 4) and the Delayer’s score in any game over ${\mathscr {C}}$ (Theorem 5).

A modal decision tree *T* for an unsatisfiable set of $\hbox {SNF}_{{mc}}$ clauses ${\mathscr {C}}$ is a tree where each vertex is labelled by some modal context $e\in \bar{{\mathscr {E}}}_{{\mathscr {C}}}^*$ and some set of clauses ${\mathscr {D}}$, and each edge is labelled by some agent $a\in {\mathscr {A}}$. Intuitively, we can think of *T* as a partial Kripke model $(W,R_{a_1},\dots , R_{a_n}, V)$ where the set *W* is the set of vertices of *T*, the relation $R_{a_i}$ is the set of $a_i$-edges of *T* for each $a_i\in {\mathscr {A}}$, and the partial valuation function *V* is s.t. if some vertex in *T* is labelled by modal context *e* then that world is *e*-accessible from the root of *T*^9^. If a vertex $\eta$ of some modal decision tree *T* is labelled by some modal context *e* then the children of $\eta$ must correspond to some query set for *e* w.r.t. ${\mathscr {D}}$.

#### Definition 25

A modal decision tree for some unsatisfiable set of $\hbox {SNF}_{{mc}}$ clauses, ${\mathscr {C}}$ is a tree *T* where:

1. Each vertex of *T* is labelled by some unique modal context $e\in \bar{{\mathscr {E}}}_{{\mathscr {C}}}^*$ and some unsatisfiable set of $\hbox {SNF}_{{mc}}$ clauses ${\mathscr {D}}$. In particular, the root is labelled by the modal context $\varepsilon$ and the set of clauses ${\mathscr {C}}$.
2. If two vertices in *T* are labelled by the modal contexts $e_1$ and $e_2$, respectively, then there is an *a*-edge from $\eta _1$ to $\eta _2$ iff $e_2=e_1(a,z)$ for some $z\in {\mathscr {X}}_{{\mathscr {C}}-}\cup {\mathscr {L}}_{{\mathscr {C}}-}$.
3. The modal context *e* and the set of clauses ${\mathscr {D}}$ labelling each vertex $\eta$ must be s.t. the set $Q_e=\{c\in \bar{{\mathscr {E}}}_{{\mathscr {C}}}\mid ec \text { labels some child of } \eta \}$ is a query set for *e* w.r.t. ${\mathscr {D}}$.
  Further, for each $c\in Q_e$, the set of clauses labelling the corresponding child of $\eta$ is ${\mathscr {D}}_{1}=\bigcup _{e_1\in \bar{{\mathscr {E}}}_{e\sqsupseteq }} {\mathscr {D}}_{e_1}\cup \bigcup _{e_1\in \bar{{\mathscr {E}}}_{ec\sqsubseteq }} {\mathscr {C}}_{e_1}\cup \bigcup _{b\in Q_{e}{\setminus }\{c\}}{\mathscr {M}}_{eb}$.

Each path *P* from the root of the tree to a given vertex specifies a partial Kripke model $M^P=(W^P,R_{a_1}^P,\dots , R_{a_n}^P, V^P)$, where $W^P=\{w_{e}\mid e\in \bar{{\mathscr {E}}}_{{\mathscr {C}}}^* \text { labels some } \eta \in P\}$, for each $i\in [n]$ $R_{a_i}=\{(\eta _{e_1},\eta _{e_2})\in P\mid (\eta _{e_1},\eta _{e_2}) \text { is an } a\text {-edge of } T\}$, and $V^P=\{V^P(w_{ec})\mid w_{ec}\in W^P\}$ where:

$$\begin{aligned} V^P(w_{ec})(x)=1 \text { if } c=(a,x) \text { or } c=(a,(x',x)). \end{aligned}$$

It is not hard to see that for each root to leaf path *P* through *T*, the partial model $M^P$ corresponds to the model constructed over the course of some two-player game over ${\mathscr {C}}$. We will further see in Proposition 4 that every $\mathbf{K }_{mc}$-Res refutation of some unsatisfiable set of $\hbox {SNF}_{{mc}}$ clauses ${\mathscr {C}}$ corresponds to some unique modal decision tree for ${\mathscr {C}}$. It is not the case however that every modal decision tree for ${\mathscr {C}}$ corresponds to a $\mathbf{K }_{mc}$-Res refutation of ${\mathscr {C}}$.

Let ${\mathscr {C}}$ be an unsatisfiable set of $\hbox {SNF}_{{mc}}$ clauses and $\pi$ be a $\mathbf{K }_{mc}$-Res refutation of ${\mathscr {C}}$. For every $e\in \bar{{\mathscr {E}}}_{{\mathscr {C}}}$ let $\pi _{e}$ denote the set of all clauses in $\pi$ with modal context $e'\in \bar{{\mathscr {E}}}_{e=}$ that are inferred using some modal rule of $\mathbf{K }_{mc}$-Res (that is, either MRES, GEN1, GEN2 or GEN3).

#### Remark 3

Throughout this section we shall assume w.l.o.g. that every clause in a $\mathbf{K }_{mc}$-Res refutation is an ancestor of $(\varepsilon :0)$. Consequently, given any unsatisfiable set of $\hbox {SNF}_{{mc}}$ clauses ${\mathscr {C}}$, any $\mathbf{K }_{mc}$-Res refutation $\pi$ of ${\mathscr {C}}$ for any $e\in \bar{{\mathscr {E}}}_{{\mathscr {C}}}^*$ s.t. $\pi _e\ne \emptyset$ the refutation $\pi$ must contain a refutation of . To see this note that any $\hbox {SNF}_{{mc}}$ clause in $\pi$ with modal context *e* must be inferred from a set of clauses whose modal contexts are either unifiable with *e* or of the form $e'c$, where $c\in \bar{{\mathscr {E}}}_{{\mathscr {C}}}$ and $e'\in \bar{{\mathscr {E}}}_{e=}$.

#### Proposition 4

Let $\pi$ be a $\mathbf{K }_{mc}$-Res refutation of some unsatisfiable set of $\hbox {SNF}_{{mc}}$ clauses ${\mathscr {C}}$. Then, we can construct a unique modal decision tree *T* that corresponds to $\pi$. Further, if *N* is the number of modal resolution steps in $\pi$ and *n* is the number of vertices *T* then $N\ge n-1$.

#### Proof

For every $e\in (\bar{{\mathscr {E}}}_{{\mathscr {C}}}{\setminus }{\mathscr {A}})^*$ s.t. $\pi _e$ is non-empty let:

$$\begin{aligned} Q_{e}=\,&\{(a,x)\in {\mathscr {A}}\times {\mathscr {X}}_{{\mathscr {C}}-} \mid (e': x'\rightarrow \Diamond _a x) \text { is used to infer some } C \in \pi _e\}\,\cup \\&\{(a,(x',x))\in {\mathscr {A}}\times {\mathscr {L}}_{{\mathscr {C}}-}\mid (e': x'\rightarrow \Diamond _a x) \text { is used to infer some } C\in \pi _e\}. \end{aligned}$$

Let the vertex set for *T* be:

$$\begin{aligned} V(T)=\{\eta _{\varepsilon }\}\cup \{\eta _{ec}\mid c \in Q_e \text { for some } e\in (\bar{{\mathscr {E}}}_{{\mathscr {C}}}{\setminus }{\mathscr {A}})^*\}. \end{aligned}$$

Further, let each $\eta _{e}\in V(T)$ be labelled by the modal context *e* and the set of $\hbox {SNF}_{{mc}}$ clauses ${\mathscr {D}}^{\eta _e}$, where:

$$\begin{aligned} {\mathscr {D}}^{\eta _{e}}={\left\{ \begin{array}{ll} {\mathscr {C}} &{} \text { if }e=\varepsilon ,\\ \bigcup _{e'\in \bar{{\mathscr {E}}}_{e_1\sqsupseteq }} {\mathscr {D}}^{\eta _{e_1}}_{e'}\cup \bigcup _{e' \in \bar{{\mathscr {E}}}_{e\sqsubseteq }} {\mathscr {C}}_{e'} \cup \bigcup _{c\in Q_{e}{\setminus }\{b\}}{\mathscr {M}}_{ec}&{}\text { if } e=e_1b \text { for some } b\in \bar{{\mathscr {E}}}_{{\mathscr {C}}}. \end{array}\right. } \end{aligned}$$

Finally for each $a\in {\mathscr {A}}$ let:

$$\begin{aligned} E_a(T)=\{(\eta _{e_1},\eta _{e_1(a,z)})\mid (a,z)\in Q_{e_1}\}, \end{aligned}$$

be the set of *a*-edges in *T*.

Clearly *T* is a tree and $|V(T)|-1=n-1\le N$. To prove that *T* is a modal decision tree for ${\mathscr {C}}$ it remains to show that each $Q_e$ is a valid query set for *e* w.r.t. ${\mathscr {D}}^{\eta _e}$. That is, we must show that each $Q_e$ satisfies conditions (a) and (b) of Definition 24.

Every negative modal clause in ${\mathscr {C}}$ with modal context $e'\in \bar{{\mathscr {E}}}_{e=}$ is in ${\mathscr {D}}^{\eta _e}$. Hence, that (a) is satisfied follows immediately from the definition of $Q_e$.

To prove that each $Q_e$ also satisfies condition (b) of the definition of a query set we will first prove that for each *e* s.t. $Q_e\ne \emptyset$, there exists some subsequence of $\pi$ which is a refutation of $\bigcup _{e_1\in \bar{{\mathscr {E}}}_{e\sqsupseteq }} {\mathscr {D}}_{e_1}^{\eta _e}\cup \pi _{e}$. This is done by induction on |*e*|. If $|e|=0$ then $e=\varepsilon$ and $\bigcup _{e_1\in \bar{{\mathscr {E}}}_{\varepsilon \sqsupseteq }} {\mathscr {D}}_{e_1}^{\eta _{\varepsilon }} ={\mathscr {C}}_{\varepsilon }$. If we further note that  it follows by Remark 3 that $\pi$ contains some refutation of $\pi _{\varepsilon }\cup {\mathscr {C}}_{\varepsilon }=\pi _{\varepsilon } \cup \bigcup _{e_1\in \bar{{\mathscr {E}}}_{\varepsilon \sqsupseteq }} {\mathscr {D}}_{e_1}^{\eta _\varepsilon }$.

Suppose $|e|>0$. Then, there exists some $e_1\in \bar{{\mathscr {E}}}_{{\mathscr {C}}}^*$ and some $b\in \bar{{\mathscr {E}}}_{{\mathscr {C}}}$ s.t. $e=e_1b$. Further, ${\mathscr {D}}^{\eta _e} =\bigcup _{e'\in \bar{{\mathscr {E}}}_{e_1\sqsupseteq }} {\mathscr {D}}_{e'}^{\eta _{e_1}}\cup \bigcup _{e'\in \bar{{\mathscr {E}}}_{e\sqsubseteq }}{\mathscr {C}}_{e'} \cup \bigcup _{c\in Q_{e_1}{\setminus }\{b\}}{\mathscr {M}}_{e_1c}$, where for each $c\in Q_{e_1}$ the set ${\mathscr {M}}_{e_1c}$ is as defined in Definition 24. By the inductive hypothesis $\pi$ contains a refutation of $\bigcup _{e'\in \bar{{\mathscr {E}}}_{e_1\sqsupseteq }} {\mathscr {D}}_{e'}^{\eta _{e_1}}\cup \mathscr {\pi }_{e_1}$. Let $\pi _{e_1}^b$ denote the subset of $\pi _e$ consisting of every clause inferred from a negative modal clause $C\in {\mathscr {C}}_{e_1b}={\mathscr {C}}_e$. It follows from the definition of $Q_{e_1}$ and Remark 3 that $\pi$ must contain a derivation of each $C\in \pi _{e_1}^b$ from ${\mathscr {C}}_{e}\cup \pi _{e}$. Hence, $\pi$ must contain a refutation of:

$$\begin{aligned} \bigcup _{e'\in \bar{{\mathscr {E}}}_{e_1\sqsupseteq }} {\mathscr {D}}_{e'}^{\eta _{e_1}} \cup (\pi _{e_1}{\setminus }\pi _{e_1}^b) \cup {\mathscr {C}}_{e} \cup \pi _{e}. \end{aligned}$$

By definition $(\pi _{e_1}{\setminus }\pi _{e_1}^b)\subseteq \bigcup _{c\in Q_{e_1}{\setminus }\{b\}}{\mathscr {M}}_{e_1c}$ and so this refutation is also a refutation of $\bigcup _{e'\in \bar{{\mathscr {E}}}_{e\sqsupseteq }} {\mathscr {D}}_{e'}^{\eta _e}\cup \pi _{e}$. Hence, we have completed our induction.

Finally to see that condition (b) is satisfied for each $Q_e$ note that as $\mathbf{K }_{mc}$-Res is sound and there exists a $\mathbf{K }_{mc}$-Res refutation of $\bigcup _{e'\in \bar{{\mathscr {E}}}_{e\sqsupseteq }} {\mathscr {D}}_{e'}^{\eta _e}\cup \pi _{e}$, this set must be unsatisfiable. It follows from the definition of $Q_e$ that $\pi _{e}\subseteq \bigcup _{c\in Q_{e}}{\mathscr {M}}_{ec}$, and so $\bigcup _{e'\in \bar{{\mathscr {E}}}_{e\sqsupseteq }} {\mathscr {D}}_{e'}^{\eta _e}\cup \bigcup _{c\in Q_{e}}{\mathscr {M}}_{ec}$ must be unsatisfiable. $\square$

In the next theorem we state the connection between the number of modal resolution steps required to refute a formula using $\mathbf{K }_{mc}$-Res and our two-player game. This connection will allow us to prove modal proof size lower bounds for $\mathbf{K }_{mc}$-Res indirectly.

#### Theorem 5

Let ${\mathscr {C}}$ be an unsatisfiable set of clauses in $\hbox {SNF}_{{mc}}$ and let $\pi$ be a $\mathbf{K }_{mc}$-Res refutation of ${\mathscr {C}}$ with *N* modal resolution steps. Then, there is a Prover strategy s.t. Delayer scores at most $\log (N+1)$ modal points.

#### Proof

Let $\pi$ be a $\mathbf{K }_{mc}$-Res refutation and let *T* be the associated modal decision tree. By Proposition 4 we have $n-1\le N$, where *n* is the number of vertices in *T* and *N* is the number of modal resolution steps in $\pi$. Hence, if we let *L*(*T*) be the set of all leaf vertices of *T* then $|L(T)|\le N+1$.

The decision tree *T* completely specifies Prover’s strategy. Recall that each vertex in *T* is labelled by some modal context and that if a vertex $\eta$ is labelled by $e\in \bar{{\mathscr {E}}}_{{\mathscr {C}}}^*$ and the set ${\mathscr {D}}$ then the set $Q_e=\{c\in \bar{{\mathscr {E}}}_{{\mathscr {C}}}\mid ec \text { labels some child of }\eta \}$ is a query for *e* w.r.t. ${\mathscr {D}}$. In particular the root vertex $\eta _0$ is labelled by the modal context $\varepsilon$ and the set ${\mathscr {C}}$, and its children correspond to some query set $Q_{\varepsilon }$ for $\varepsilon$ w.r.t. ${\mathscr {C}}$. Prover queries the set $Q_{\varepsilon }$. If $Q_{\varepsilon }=\emptyset$ then the game ends. Otherwise, Delayer gives each $c\in Q_{\varepsilon }$ a weight $p_c$ and Prover chooses some $c=(a_c,z)\in Q_{\varepsilon }$ with probability $p_{c}$, sets:

$$\begin{aligned} e^2&=c, \;\; s^2=\log \frac{1}{p_c},\;\;R_{a}^{2}= {\left\{ \begin{array}{ll} R_{a}^{1}\cup \{(w_{\varepsilon }, w_{c})\} &{} \text {if } a=a_{c},\\ R_a^{1} &{} \text {otherwise}, \end{array}\right. } \\ W^{2}&=W^{1}\cup \{w_{c}\} \text { and } V^{2}(w_{c})(x)= 1 \text { if } x=z \text { or } (x',x)=z \text { for some } x'\in {\mathscr {X}}_{{\mathscr {C}}}. \end{aligned}$$

and moves along the corresponding edge of *T* to the vertex labelled by *c*.

At the next round Prover queries the set $Q_{c}$ corresponding to the children of this new vertex and proceeds as above. Continuing in this manner will result in a root to leaf walk on *T*. Note that the set of all possible such walks is in bijection with the set of leaves of *T*.

Let $q_{D,\lambda }$ denote the probability of the game ending at leaf $\lambda \in L(T)$ when played with a fixed Delayer *D*. Let $\pi _D$ be the probability distribution over the leaves of *T*. If the game ends at leaf $\lambda$ then *D* scores exactly $\log \frac{1}{q_{D,\lambda }}$ points.

To see this consider a fixed leaf $\lambda$ and the unique path *P* from the root of *T* to $\lambda$. The modal context of the *i*th vertex in *P* is $e^{i}$. Hence, the probability of reaching $\lambda$ is:

$$\begin{aligned} q_{D,\lambda }=q_1q_2\dots q_m, \end{aligned}$$

where $q_j$ is the probability of choosing $c_j$ from $Q_{e^{i}}$. The score at the end of the game is:

$$\begin{aligned} \sum _{j=1}^{m}\log \frac{1}{q_j}=\log \frac{1}{\prod _{i=j}^{m} q_{j}}=\log \frac{1}{q_{D,\lambda }}, \end{aligned}$$

and the expected score of the Delayer is:

$$\begin{aligned} \sum _{\lambda \in L(T)} q_{D,\lambda }\log \frac{1}{q_{D,\lambda }}=H(\pi _D), \end{aligned}$$

which is exactly the Shannon entropy of $\pi _D$. The entropy is maximal when the probability distribution considered is the uniform distribution, hence as the support of $\pi _D$ has size at most |*L*(*T*)| it follows that $H(\pi _D)\le \log |L(T)|\le \log (N+1)$. $\square$

The above theorem allows us to prove lower bounds on the number of modal resolution steps needed to refute some unsatisfiable set of $\hbox {SNF}_{{mc}}$ clauses ${\mathscr {C}}$ using $\mathbf{K }_{mc}$-Res. Such lower bounds are proved indirectly by first proving a lower bound *f*(*n*), where *n* is the size of ${\mathscr {C}}$, on the Delayers score for any game played on a given unsatisfiable set of $\hbox {SNF}_{{mc}}$ clauses ${\mathscr {C}}$. It follows from the above theorem that $2^{f(n)}$ is a lower bound for the number of modal resolution steps, *N* required to refute ${\mathscr {C}}$, hence if $2^{f(n)}$ is superpolynomial then we have proved a superpolynomial lower bound for *N*.

## An Exponential Lower Bound for the Modal Pigeonhole Principle

The pigeonhole principle with *m* pigeons and *n* pigeonholes states that whenever $m>n$ there is no $1-1$ map from the pigeons to the pigeonholes. This can be formulated as a propositional formula as follows:

$$\begin{aligned} PHP_n^m=\bigwedge _{i\in [n]}\bigvee _{j\in [m]}p_{i,j}\wedge \bigwedge _{1\le i<i'\le n}\bigwedge _{j\in [m]}(\lnot p_{i,j}\vee \lnot p_{i',j}). \end{aligned}$$

Intuitively, the propositional variable $p_{i,j}$ denotes that the *i*th pigeon is in the *j*th pigeonhole, hence the above formula says that each pigeon is in a pigeonhole and that no pigeonhole contains more than one pigeon. Clearly whenever $m>n$ the formula $PHP_n^m$ must be unsatisfiable. The propositional pigeonhole principle is known to be hard for propositional resolution [38].

We can formulate the pigeonhole principle as a modal formula with modal depth *m* over the set of agents ${\mathscr {A}}=\{a\}$ and the set of variables $\{l_1,\dots , l_n\}$.

### Definition 26

($\mathbf {MPHP}_n^m$) Let $P_{i}=\Box ^{i-1} (\bigvee _{j=1}^{n} \Diamond l_j)$ for every $1\le i\le m$ and $H_{i,i'}^j=\Box ^i \lnot l_j\vee \Box ^{i'}\lnot l_j$ for every $1\le j\le n$ and $1\le i<i'\le m$. We define:

$$\begin{aligned} \mathbf {MPHP}_{n}^m=\bigwedge _{i} P_i\wedge \bigwedge _{j}\bigwedge _{i'\ne i}\bigwedge _{i} H_{i,i'}^{j}. \end{aligned}$$

Note that as $\mathbf {MPHP}_{n}^m$ is a modal formula over just a single agent we have omitted the subscripts from our modal operators. Further, $\Box ^{i}$ denotes *i* successive $\Box _a$ operators.

Intuitively pigeon *i* is in pigeonhole *j* only if $\Diamond ^i l_j=1$, where $\Diamond ^i$ denotes *i* successive $\Diamond _a$ operators. Hence, $P_i$ says that pigeon *i* occupies at least one pigeonhole and $H_{i,i'}^j$ says that no two pigeons occupy the same hole. Clearly whenever $m>n$ (that is, there are more pigeons than pigeonholes) $\mathbf {MPHP}_n^m$ is unsatisfiable.

We can easily convert $\mathbf {MPHP}_n^m$ into the following set of $\hbox {SNF}_{{mc}}$ formulas:

$$\begin{aligned} \mathscr {MPHP}_n^{m}=x_{\varepsilon }\wedge \bigwedge _{i} {\hat{P}}_i\wedge \bigwedge _{j}\bigwedge _{i'\ne i}\bigwedge _{i} {\hat{H}}_{i,i'}^{j} \end{aligned}$$

where $i,i'\in [m]$, $j\in [n]$ and for all $i_1\in \{2,\dots , m\}$, $i,i'$ and *j*:

$$\begin{aligned}&\begin{aligned} {\hat{P}}_1 ={}&(\varepsilon :\lnot x_{\varepsilon }\vee x^{1}_{1}\vee y^{1}_{1})\;\wedge \\&\bigwedge _{k_1=2}^{n-2} (\varepsilon : \lnot x^{1}_{k_1-1}\vee x^{1}_{k_1}\vee y^{1}_{k_1})\wedge (\varepsilon : \lnot x^{1}_{n-2}\vee y^{1}_{n-1}\vee y^{1}_{n})\wedge \bigwedge _{k_2=1}^n(\varepsilon : y^{1}_{k_2}\rightarrow \Diamond l_{k_2}), \end{aligned}\\&\begin{aligned} {\hat{P}}_{i_1} ={}&( \varepsilon : x_{\varepsilon }\rightarrow \Box z^{i_1}_{1})\wedge \bigwedge _{k_1=1}^{i_1-2} \left( a^{k_1}: z^{i_1}_{k_1}\rightarrow \Box z^{i_1}_{k_1+1}\right) \wedge \\ {}&(a^{i_1-1}:\lnot z^{i_1}_{i_1}\vee x^{i_1}_{1}\vee y^{i_1}_{1})\wedge \bigwedge _{k_2=2}^{n-2}\left( a^{i_1-1}:\lnot x^{i_1}_{k_2-1}\vee x^{i_1}_{k_2}\vee y^{i_1}_{k_2}\right) \wedge \\ {}&(a^{i_1-1}:\lnot x^{i_1}_{n-2}\vee y^{i_1}_{n-1}\vee y^{i_1}_{n}) \wedge \bigwedge _{k_3=1}^n(a^{i_1-1}: y^{i_1}_{k_3}\rightarrow \Diamond l_{k_3}), \end{aligned}\\&{\hat{H}}_{i,i'}^j = (\varepsilon :\lnot x_{\varepsilon }\vee x_{i,i',1}^{j}\vee y_{i,i',1}^{j})\wedge \bigwedge _{k_1=1}^{i-1}\left( a^{k_1-1}: x_{i,i',k_1}^{j}\rightarrow \Box x_{i,i',k_1+1}^{j}\right) \wedge \\&(a^{i-1}: x_{i,i',i}^{j}\rightarrow \Box \lnot l_j) \wedge \bigwedge _{k_2=1}^{i'-1}\left( a^{k_2-1}: y_{i,i',k_2}^{j} \rightarrow \Box y_{i,i',k_2+1}^{j}\right) \wedge (a^{i'-1}: y_{i,i',i'}^{j} \rightarrow \Box \lnot l_j). \end{aligned}$$

Note that we have written our formula as a conjunction of clauses, however we can equivalently think of it as a set of clauses. Hence, in the remainder of this section we will use set notation.

In the following theorem we prove that the number of modal resolution steps used in any $\mathbf{K }_{mc}$-Res refutation of $\mathscr {MPHP}_{n}^m$ is superpolynomial with respect to *n*. To obtain this lower bound we show that if the two-player game defined in Sect. 6.2 is played on $\mathscr {MPHP}_{n}^m$ then Delayer can play according to a certain strategy which ensures that he always scores at least $\log (n!)$ points, no matter what strategy Prover adopts. Recall Theorem 5 states that, if there exists a refutation of $\mathscr {MPHP}_n^m$ with *N* modal resolution steps then Prover can ensure Delayer score never exceeds $\log (N+1)$ points. Hence, our lower bound follows.

### Theorem 6

Any $\mathbf{K }_{mc}$-Res refutation of $\mathscr {MPHP}^m_n$ has at least $n!-1$ modal steps.

### Proof

Let ${\mathscr {C}}=\mathscr {MPHP}_{n}^m$. Suppose a Prover and a Delayer play the game defined in Sect. 6.2 on ${\mathscr {C}}$. By definition, at the beginning of the game we have the modal context $e^1=\varepsilon$, the set of clauses ${\mathscr {D}}^1={\mathscr {C}}$ and the pointed model $\langle M^1,w_{\varepsilon }$ where $M^1=(W^1, R^1, V^1)$, $W^1=\{w_{\varepsilon }\}$, $R^1=\emptyset$ and $V^1(w_{\varepsilon })(x_{\varepsilon })=1$. At the *k*th round of the game Prover fixes some query set $Q_{e^{k}}$ for $e^{k}$ w.r.t. ${\mathscr {D}}^{k}$ and then adds a world that is $e^{k}b$-accessible from $w_{\varepsilon }$ to the model, where $b\in Q_{e^{k}}$.

Let $Q_{e^k}^{max}=\{(a,( y_{j}^{k},l_{j}))\mid j\in [n]\}$. As $N_{e^k}=\{(a^{k-1}: y_j^k\rightarrow \Diamond l_j)\mid j\in [n]\}$ it follows by condition (a) of Definition 24 that every query set for $e^{k}$ is a subset of $Q_{e^k}^{max}$. If Prover chooses to add a world that is $e^{k}(a,(y_j^k,l_j))$-accessible from $w_{\varepsilon }$ to the model then $(M,w_{\varepsilon })\models \Diamond ^k l_j$. Hence, intuitively at the *k*th round of the game Prover chooses some pigeonhole for the *k*th pigeon to occupy. If we let $A_k=\{(a,(y_{j}^k, l_{j})\mid (a, (y_{j}^{k_1},l_{j}))\vartriangleleft e^{k}, \; k_1\in [k-1],\; j\in [n]\}$ then $A_k$ is the set of pigeonholes occupied by the first $k-1$ pigeons.

We will now give Delayer’s strategy for the first *n* rounds of the game. If Prover queries some set $Q_{e^{k}}$ at the *k*th round of the game then for each $b=(a,(y_j^k,l_j))\in Q_{e^{k}}$ Delayer sets the weights as follows:

$$\begin{aligned}&p_b=\frac{1}{|Q_{e^{k}}|-|A_k|},&\text {if } b\not \in A_k,\\&p_b=0,&\text {otherwise.} \end{aligned}$$

At each round Delayer forces Prover to put the *k*th pigeon into some unoccupied pigeonhole. Obviously this strategy can only be followed if $|Q_{e^{k}}|-|A_k|>0$. Hence, in order to prove our lower bound we need the following claim.

### Claim

For every $k\in [n]$ the set $Q_{e^{k}}= Q_{e^k}^{max}$.

We have already seen that $Q_{e^k}\subseteq Q_{e^k}^{max}$. To see why $Q_{e^k}\supseteq Q_{e^k}^{max}$ we can think of each query set $Q_{e^k}$ as a set of candidate pigeonholes for the *k*th pigeon. By Proposition 2 the set $Q_{e^k}$ must contain every pigeonhole that can possibly be occupied by pigeon *k*, while satisfying $\bigcup _{e\in \bar{{\mathscr {E}}}_{e^k\sqsupseteq }}{\mathscr {D}}_e^k$. This set is satisfied by any model corresponding to an assignment of pigeons to pigeonholes where for each $i\in [k-1]$ the *i*th pigeon is put into the pigeonhole specified to by the *i*th symbol of $e^k$, and the *k*th pigeon is just in some pigeonhole. Hence, as there is no restriction on which pigeonhole is occupied by pigeon *k* we have $Q_{e^k}\supseteq Q_{e^k}^{max}$.

Before giving a formal proof of our claim we will explain how it allows us to prove our lower bound. By the above claim $|Q_{e^{k}}|=n$ and so for each *k* we have $|Q_{e^k}|-|A_k|=n-(k-1)$. Hence, Delayer can follow the above strategy for the first *n* rounds. It follows that Delayers score at the end of the *n*th round will be:

$$\begin{aligned} s^{n}=\sum _{k=1}^{n}\left( \log (n+1-k)\right) =\log \left( \prod _{k=1}^{n} (n+1-k)\right) =\log (n!). \end{aligned}$$

As Delayer scores at least 0 at each round his final score must be $\ge s^{n}$ and so by Theorem 5 any $\mathbf{K }_{mc}$-Res refutation of $\mathscr {MPHP}_n^m$ contains at least $2^{s^{n}}-1=n!-1$ modal steps.

### Proof of claim

We will now give a formal proof of our claim. To prove that $Q_{e^k}\supseteq Q_{e^k}^{max}$ it suffices to show that for every $b\in Q_{e^k}^{max}$ there exists some model $M=(W,R,V)$ and some world $w_{\varepsilon }\in W$ s.t. $(M,w_{\varepsilon })\models \bigcup _{e_1\in \bar{{\mathscr {E}}}_{e^k\sqsupseteq }}{\mathscr {D}}^k_{e_1} \cup \bigcup _{c\in Q_{e^k}^{max}{\setminus }\{b\}}{\mathscr {M}}_{ec}$, where each ${\mathscr {M}}_{ec}$ is as defined in part (b) of Definition 24.

In fact we prove will a slightly stronger result. Namely that for every $e^{k}$ and every $b\in Q_{e^k}^{max}$ there exists a model $M=(W,R,V)$ where:

1 $$\begin{aligned} W=\{w_{e}\mid e\sqsubseteq e^{k}\},\;\;\;\;R=\{(w_{e},w_{ec}) \mid w_{e},w_{ec}\in W \text { and } c\in \bar{{\mathscr {E}}}_{{\mathscr {C}}} \}, \end{aligned}$$

2 $$\begin{aligned} V(w_e)(l_{j})={\left\{ \begin{array}{ll} 1 &{} \text {if } j=k_2 \text { and } e=e'(a,(y_{k_2}^{k_1}, l_{k_2})) \text { for some } e'\in \bar{{\mathscr {E}}}_{{\mathscr {C}}}^*,\\ 0 &{} \text {if } j\ne k_2 \text { and } e=e'(a,(y_{k_2}^{k_1}, l_{k_2})) \text { for some } e'\in \bar{{\mathscr {E}}}_{{\mathscr {C}}}^*, \end{array}\right. } \end{aligned}$$

for all $w_{e}\in W$ and all $j,k_1,k_2\in [n]$. Further, $(M,w_{\varepsilon })\models \bigcup _{e_1\in \bar{{\mathscr {E}}}_{e^k\sqsupseteq }}{\mathscr {D}}^k_{e_1}\cup \bigcup _{c\in Q_{e^k}^{max}{\setminus }\{b\}}{\mathscr {M}}_{ec}$. To prove this we use induction on *k*.

If $k=1$ then $e^1=\varepsilon$. Let $b=(a,(y_{p}^1,l_{p}))\in Q_{\varepsilon }^{max}$ and let $M=(W,R_a,V)$ where:

$$\begin{aligned}&W=\{w_{\varepsilon } \},\;\;\; R_a=\emptyset ,\;\;\;V(w_{\varepsilon })(x_{\varepsilon })=1, \\&\quad V(w_{\varepsilon })(y_{j}^{1})={\left\{ \begin{array}{ll} 1 &{} \text {if } j=p,\\ 0 &{} \text {if } j\ne p, \end{array}\right. }\; \text { and }\; V(w_{\varepsilon })(x_{j}^{1})={\left\{ \begin{array}{ll} 1 &{} \text {if } j<p,\\ 0 &{} \text {if } j\ge p, \end{array}\right. } \end{aligned}$$

for all $j\in [n]$. Clearly $(M,w_{\varepsilon })\models \bigcup _{e\in \bar{{\mathscr {E}}}_{\varepsilon \sqsupseteq }}{\mathscr {D}}^1_e={\mathscr {C}}_{\varepsilon }$. Further, for each $c=(a,(y_j^1,l_j))\in Q_{e^1}^{max}$ the set ${\mathscr {M}}_{c}$ contains only clauses that can be inferred by applying some modal rule of $\mathbf{K }_{mc}$-Res to some set of clauses containing $(\varepsilon :y_{j}^{1}\rightarrow \Diamond l_{j})$. Hence, every clause in ${\mathscr {M}}_{c}$ is of the form $(\varepsilon :A\vee \lnot y_{j})$, where *A* is a propositional clause and so $(M,w_{\varepsilon })\models \bigcup _{c\in Q_{\varepsilon }^{max}{\setminus }\{b\}}{\mathscr {M}}_{c}$.

Suppose $k>1$. Delayer’s strategy ensures that $e^{k}=e^{k-1}b_1$ for some $b_1\in Q_{e^{k-1}}^{max}{\setminus } A_{k-1}$. Hence, by the inductive hypothesis there exists a partial model $M'=(W',R',V')$ where:

$$\begin{aligned} W'=\{w_{e}\mid e\sqsubseteq e^{k-1}\},\;\;R'=\{(w_{e},w_{ey}) \mid w_{e},w_{ec}\in W \text { and } c\in \bar{{\mathscr {E}}}_{{\mathscr {C}}} \}\\ V'(w_e)(l_{j})={\left\{ \begin{array}{ll} 1 &{} \text {if } j=j_1 \text { and } e=e'(a,(y_{j_1}^{k_1}, l_{j_1})) \text { for some } e'\in \bar{{\mathscr {E}}}_{{\mathscr {C}}}^*,\\ 0 &{} \text {if } j\ne j_1 \text { and } e=e'(a,(y_{j_1}^{k_1}, l_{j_1})) \text { for some } e'\in \bar{{\mathscr {E}}}_{{\mathscr {C}}}^*, \end{array}\right. } \end{aligned}$$

for all $w_{e}\in W$ and all $j\in [n]$, and $(M',w_{\varepsilon })\models \bigcup _{e_1\in \bar{{\mathscr {E}}}_{e^{k-1} \sqsupseteq }}{\mathscr {D}}^{k-1}_{e_1}\bigcup _{c\in Q_{e^{k-1}}^{max}{\setminus }\{b_1\}}{\mathscr {M}}_{e^{k-1}c}$.

For each $b_2=(a,(y_{p}^k,l_{p}))\in Q_{e^k}^{max}$ we can construct a model *M*, whose worlds, relations and valuations are as in Equations (1) and (2), and which satisfies $\bigcup _{e_1\in \bar{{\mathscr {E}}}_{e^k\sqsupseteq }}{\mathscr {D}}^k_{e_1} \cup \bigcup _{c\in Q_{e^{k}}^{max}{\setminus }\{b_2\}}{\mathscr {M}}_{e^{k-1}c}$ at $w_{\varepsilon }$ as follows. Let $M=(W,R,V)$ where:

$$\begin{aligned} W&=W'\cup \{w_{e^{k}}\},\;\; R=R'\cup \{(w_{e^{k-1}},w_{e^{k}})\},\\ V(w)(x)&=V'(w)(x) \text { for every } w\in W'\; \text { and }\; V(w_{e^{k}})(l_{j})={\left\{ \begin{array}{ll} 1 &{} \text {if } b_2=(a,(y_{j}^k,l_{j})),\\ 0 &{} \text { otherwise.} \end{array}\right. } \end{aligned}$$

Clearly *M* is a model of the required form. Further, as *M* is an extension of $M'$ we have $(M,w_{\varepsilon })\models \bigcup _{e_1\in \bar{{\mathscr {E}}}_{e^{k-1} \sqsupseteq }}{\mathscr {D}}^{k-1}_{e_1}\bigcup _{c\in Q_{e^{k-1}}^{max}{\setminus }\{b_1\}}{\mathscr {M}}_{e^{k-1}c}$. Hence, to show that $Q_{e^k}^{max}{\setminus } \{b_2\}$ is not a query set for $e^k$ w.r.t. ${\mathscr {D}}^k$ all we have to do is show that $(M,w_{\varepsilon })\models {\mathscr {C}}_{e^k}\cup \bigcup _{c\in Q_{e^{k}}^{max}{\setminus }\{b_2\}}{\mathscr {M}}_{e^{k}c}$.

Recall that for each $c=(a,(y_{j}^k,l_j))\in Q_{e^k}^{max}$ the set ${\mathscr {M}}_{ec}$ contains only clauses of the form $(A\vee \lnot y_{j}^k)$, where $A\in \mathscr {CL}$. Hence, to ensure that $(M, w_{\varepsilon })\models \bigcup _{c\in Q_{e^{k}}^{max}{\setminus }\{b_2\}}{\mathscr {M}}_{e^kc}$ we let:

$$\begin{aligned} V(w_{e^{k}})(y_{j}^{k})={\left\{ \begin{array}{ll} 1 &{} \text {if } j=p,\\ 0 &{} \text {if } j\ne p. \end{array}\right. } \end{aligned}$$

Note that the set ${\mathscr {C}}_{e^{k}}$ consists of every literal clause in ${\mathscr {C}}$ with modal context $a^{k-1}$, every positive modal clauses in ${\mathscr {C}}$ with modal context $a^{k-2}$ and the negative modal clause $(a^{k-2}:y_q^{k-1}\rightarrow \Diamond l_q)$, where *q* s.t. $b_1=(a,(y_{q}^{k-1}, l_q))$. As $V(w_{e^{k}})(l_j)=1$ iff $j=q$ clearly $(M,w_{\varepsilon })\models (a^{k-2}:y_q^{k-1}\rightarrow \Diamond l_q)$. If we further let:

$$\begin{aligned} V(w_{e^{k}})(z_{k-1}^{i_1})=1\;\text { and }\; V(w_{e^{k}})(x_{j}^{k})={\left\{ \begin{array}{ll} 1 &{} \text {if } j<p,\\ 0 &{} \text {if } j\ge p, \end{array}\right. } \end{aligned}$$

for all $k<i_1\le n$ and all *j* then it is not hard to see that every positive modal clause of the form $(a^{k-2}: z_{k-2}^{i'}\rightarrow \Box z_{k-1}^{i'})$ and every literal clause in ${\hat{P}}_{k-1}$ is satisfied at $w_{\varepsilon }$ in *M*.

We have now shown that $(M,w_{\varepsilon })\models {\mathscr {C}}_{e^{k}}\cap \bigcup _{i}{\hat{P}}_i$. It remains is to show that $(M,w_{\varepsilon })\models {\mathscr {C}}_{e^k}\cap \bigcup _{i\ne i', j}{\hat{H}}_{i,i'}^j$. The set ${\mathscr {C}}_{e^k}\cap \bigcup _{i\ne i, j}{\hat{H}}_{i,i'}^j$ consists of all clauses of the following forms: 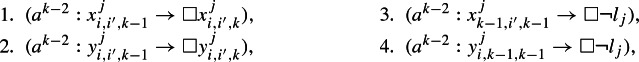


where $i< i'\in [m]$ and $j\in [n]$. Every clause of the form 1 and 2 can be satisfied at $w_{\varepsilon }$ in *M* by letting:

$$\begin{aligned} V(w_{e^k})(x_{i,i',k}^j)=1 \text { and } V(w_{e^k})(y_{i,i',k}^j)=1. \end{aligned}$$

However, clauses of the form 3 and 4 do not contain any unassigned variables, so we cannot simply extend *V* to ensure that they are satisfied at $w_{\varepsilon }$ in *M*. A clause $(a^{k-2}: x_{k-1,i',k-1}^{j}\rightarrow \Box \lnot l_j)$ is not satisfied at $w_{\varepsilon }$ in *M* iff $V(w_{e^{k-1}})(x_{k-1,i',k-1}^j)=0$ and $V(w_{e^k})(l_j)=1$. As $V(w_{e^k})(l_j)=0$ for all $j\ne q$ this can only be the case if $j=q$. Hence, to ensure that every such clause is satisfied we set:

$$\begin{aligned} V(w_{e^{i_1}})(x_{k-1,i',i_1}^{j})=0 \text { and } V(w_{e^{i_1}})(y_{k-1,i',i_1}^{j})=1 \text { for all } i_1< k, \end{aligned}$$

Note that every clause containing these variables is in $H_{i,i'}^j$. By inspection, we can easily see that changing the assignments of these variables as above does not change the truth valuation of any clause in ${\mathscr {C}}_{e^k}\cap H_{i,i'}^j$. Similarly to ensure that every clause of the form $(a^{k-2}: y_{i,k-1,k-1}^{j}\rightarrow \Box \lnot l_j)$ is satisfied at $w_{\varepsilon }$ in *M* we set:

$$\begin{aligned} V(w_{e^{i_1}})(x_{i,k-1,i_1}^{j})=1 \text { and } V(w_{e^{i_1}})(y_{i,k-1,i_1}^{j})=0 \text { for all } i_1< k \end{aligned}$$

Note that this will not cause the clause $(a^{i-2}: x_{i,k-1,i}^j\rightarrow \Box \lnot l_j)$ to be falsified as Delayer’s strategy ensures that $e^k$ contains no repeated pigeonholes and so $V(w_{e^i})(l_j)=0$. Hence, as above changing the assignments of these variables will not change the truth valuation of any clause in ${\mathscr {C}}_{e^k}\cap H_{i,i'}^j$.

This concludes the proof of our claim, and so the proof of the theorem. $\square$

## Comparing Modal Frege Systems with Modal Resolution Systems

In proof complexity, Frege systems are among the most studied proof systems for propositional logic.

### Definition 27

A *Frege system* for propositional logic is a line-based proof system *P* consisting of a finite set of inference rules and axioms of the form $\phi _1,\dots , \phi _k\vdash _P \phi$ and $\vdash _P\phi$, respectively, where $\phi _1,\dots ,\phi _k,\phi$ are propositional formulas. Further, *P* must be sound and strongly complete.

One way to prove that a propositional formula $\phi$ is a tautology using a Frege system is to refute its negation. That is, to derive a formula of the form $\lnot \phi \rightarrow 0$.

**Fig. 4:**
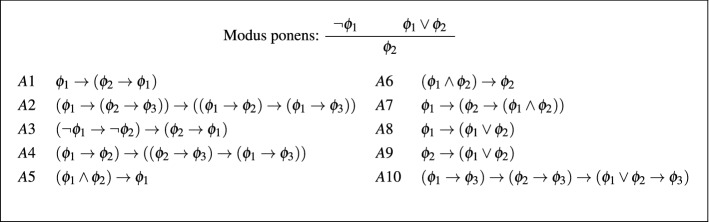
A propositional Frege system

An example of a propositional Frege system is given in Fig. 4. It was shown in [31] that every Frege system is *p*-equivalent hence we can take this system (or any other) to be the canonical Frege system.

### Definition 28

Let *P* be a Frege system. We say that a rule $\phi _1,\dots ,\phi _z\vdash \phi$ of *P* is *sound* if every model which satisfies $\phi _1,\dots ,\phi _z$ also satisfies $\phi$.

The resolution rule is sound and so can be taken as a rule of any Frege system and so clearly Frege *p*-simulates propositional resolution. Furthermore, there exists an exponential separation between Frege and propositional resolution [28]. That is, there exist propositional formulas that are known to require exponential-sized proofs for propositional resolution and polynomial-sized proofs for Frege.

### Definition 29

An *extended Frege* system is a Frege system with the additional axiom:

$$\begin{aligned} p\leftrightarrow \phi , \end{aligned}$$

where *p* is a new propositional variable (called an extension variable), that does not appear in $\phi$, any previously derived formulas or the final formula of the proof. We call this axiom the *extension axiom*.

While extended Frege obviously *p*-simulates Frege, there is as of yet no known separation between these systems. In fact there are currently no propositional formulas that have been shown to require super-polynomial size proofs in Frege.

Given any propositional Frege system we can obtain a Frege system for the modal logic $\mathbf{K }_n$ by adding the following rules, for every $a\in {\mathscr {A}}$: 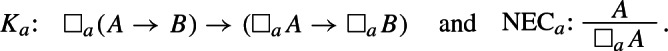


Further, an *extended Frege system for* $\mathbf{K }_n$ can be obtained by adding the extension axiom to any $\mathbf{K }_n$-Frege system.

### Definition 30

[42] We say that a $\mathbf{K }_n$-Frege system *P* is *standard* if every formula $\phi$ for which $\phi _1,\dots ,\phi _k\vdash _P \phi$ is in the closure of $\mathbf{K }_n\cup \{\phi _1,\dots ,\phi _k\}$ under MP and $\hbox {NEC}_a$ for every $a\in {\mathscr {A}}$.

We can extend Definition 28 to $\mathbf{K }_{n}$-Frege systems by saying that a rule, $\phi _1,\dots ,\phi _z$ is sound iff whenever $\phi _1,\dots ,\phi _z$ are satisfied at some world *w* in some Kripke model *M*, so is $\phi$. Clearly a $\mathbf{K }_n$-Frege system can be non-standard only if it contains some rule, other than $\hbox {NEC}_a$, which is not sound. Every standard $\mathbf{K }_n$-Frege system *p*-simulates every other standard $\mathbf{K }_n$-Frege system [42]. The analogous statement is not known to hold for non-standard $\mathbf{K }_n$-Frege systems, hence in this paper we only consider standard $\mathbf{K }_n$-Frege systems.

### Lemma 6

$\mathbf{K }_n$-Frege *p*-simulates $\mathbf{K }_{mc}$-Res.

### Proof

Each of the rules of $\mathbf{K }_{mc}$-Res is sound. Hence, the lemma follows immediately. $\square$

### Separation of $\mathbf{K }_n$-Frege and $\mathbf{K }_{mc}$-Res

It was proved by Buss in [28] that there exist polynomial-sized Frege refutations of the propositional pigeonhole principle with $m>n$ pigeons. Buss’ proof relies on the fact that Frege systems can count efficiently. The idea behind the proof is as follows. If we assume that the pigeonhole principle holds for some $m>n$ and count the number of holes that are occupied by the first $n+1$ pigeons then we can construct a polynomial-sized Frege derivation of some formula encoding that this number is greater than *n*. However, as there are only *n* pigeonholes, we can also construct polynomial-sized Frege derivation of a formula encoding that the number of occupied holes is less than or equal to *n*, leading to a contradiction. A very similar proof can be used to show that there exists a polynomial-sized ${\mathbf {K}}_n$-Frege proof of the modal pigeonhole principle. We will give a sketch of Buss’ proof of the pigeonhole principle, highlighting the steps that make explicit use of $PHP_n^m$.

#### Theorem 7

[28] There exist polynomial-sized Frege refutations of $PHP_n^m$, where $m>n$.

#### Proof

(Sketch) The proof has two parts. First we show that there exists a polynomial-sized extended Frege derivation of 0 from $PHP_n^m$. We will then show that this extended Frege derivation can be used to obtain a polynomial-sized Frege derivation of 0 from $PHP_n^m$.

The extended Frege refutation of $PHP_n^m$ is obtained as follows. First, for each $i\in [m]$ and $j\in [n]$ we introduce an extension variable $r_j^i$ which abbreviates the formula $\bigvee _{k\in [i]}p_{k,j}$. Clearly $r_j^i$ is true iff one of the first *i* pigeons occupies pigeonhole *j*. Hence, the number of $r_j^i$’s that are true in a given assignment is equal to the number of pigeonholes occupied by the first *i* pigeons. If we assume w.l.o.g. that *n* is a power of 2 then for each *i* we can define $\log n$ formula’s $a^{i,1},\dots ,a^{i,\log n}$ (henceforth denoted by the vector $\vec {a}^i$) which encode the number of $r_{j}^i$’s which are true in a given model. We denote the number encoded by $\vec {a}^{i}$ as $A^i$.

Let $\phi _i=(\bigwedge _{j\in [n]} (r_j^i\rightarrow r_j^{i+1})\wedge \bigvee _{j\in [n]}(r_j^{i+1}\wedge \lnot r_{j}^i))$. It is not hard to see from the definition of $r_j^1$ that there exists a polynomial-sized Frege proof of $PHP_n^m \rightarrow \bigvee _{j\in [n]} r_j^1$. Further, given $\bigvee _{j\in [n]} r_j^1$ there exists a polynomial-sized Frege derivation of a formula encoding that $0<A^1$. Similarly, for each $i\in [m]$ there exists a straightforward polynomial-sized Frege proof of $PHP_n^m\rightarrow \phi _i$ and, for each *i* given $\phi _i$ there exists a polynomial-sized Frege derivation of a propositional formula encoding that $A^i<A^{i+1}$. Finally these proofs can be combined to obtain polynomial-sized proofs of a formula encoding that $n<A^{n+1}$ from $\bigwedge _{i\in [m]}\phi _i\wedge \bigvee _{j\in [n]} r_{j}^1$ (and so from $PHP_n^m$).

There further exists a polynomial-sized Frege proof of a formula which encodes that $n\le A^n$. Hence, there exists a polynomial-sized Frege derivation of a formula encoding that $n<n$ from $PHP_n^m$. As $n=n$ this formula must be false and so we have a polynomial-sized Frege refutation of $PHP_n^m$.

For every *i*, each formula in $\vec {a}^i$ can be defined so that it has size polynomial in that of the largest propositional formula abbreviated by any $r_j^i$. Hence, replacing all the extension variables in the extended Frege proof of $PHP_m^n\rightarrow 0$ with the formulas they abbreviate yields a polynomial-sized Frege refutation of $PHP_n^m$. $\square$

#### Theorem 8

There exists a polynomial-sized $\mathbf{K }_n$-Frege refutation of $MPHP_n^m$, where $m>n$.

#### Proof

(Sketch) Note that the only parts of the extended Frege refutation of $PHP_n^m$ that depend on the formula itself are the proofs of $PHP_n^m \rightarrow \bigvee _{j\in [n]} r_j^1$ and $PHP^m_n \rightarrow \bigwedge _{i\in [m]}\phi _i$. Hence, to show that there exists a polynomial-sized extended $\mathbf{K }_n$-Frege refutation of $MPHP_n^m$ it suffices to show that we there exist polynomial-sized $\mathbf{K }_n$-Frege proofs of $MPHP_n^m\rightarrow \bigvee _{j\in [n]} r_j^1$ and $MPHP_n^m\rightarrow \bigwedge _{i\in [n-1]} \phi _i$ for some suitable choice of extension variables $r_j^i$.

Let $r_{j}^i\leftrightarrow \bigvee _{k\in [i]}\Diamond ^k l_j$. Clearly we can derive $MPHP_n^m\rightarrow \bigvee _{j\in [n]} r_j^1$. Further, for each $j\in [n]$ and $i\in [m-1]$ there exists a simple Frege proof of $r_j^i\rightarrow r_j^{i+1}$. It remains to show that for each $i\in [m-1]$ there exists a polynomial-sized Frege proof of $\lnot MPHP_n^m \rightarrow \bigvee _{j\in [n]} (r_j^{i+1}\wedge \lnot r_{j}^i))$. To see this first note that there exists a polynomial-sized Frege proof of $(\Diamond ^{i+1} l_{j}\wedge \bigwedge _{k\in [i]}\lnot \Diamond ^{k} l_j) \rightarrow r_j^{i+1}\wedge \lnot r_j^i$. As the formulas $\Diamond \psi _1\wedge \Box \psi _2\rightarrow \Diamond \psi _2$ and $\Diamond \psi _1\vee \Diamond \psi _2\rightarrow \Diamond (\psi _1\vee \psi _2)$ are theorems of $\mathbf{K }_n$ we can assume w.l.o.g. that they are axioms of our $\mathbf{K }_n$-Frege system. Hence, we can easily obtain a polynomial-sized $\mathbf{K }_n$-Frege proof of $P_{i+1}\rightarrow \bigvee _{j\in [n]}\Diamond ^{i+1} l_j$. Given this it is not hard to see that $P_{i+1}\wedge \bigwedge _{j\in [n]}H_{i,i+1}^j\rightarrow ( \Diamond ^{i+1} l_{j}\wedge \bigwedge _{k\in [i]}\lnot \Diamond ^{k} l_j)$. Putting these proofs together, we obtain a polynomial-sized proof of $MPHP_n^m\rightarrow \bigvee _{j\in [n]} (r_j^{m+1}\wedge \lnot r_{j}^m))$. Hence, we have shown that $MPHP_n^m\rightarrow \bigwedge _{i\in [n-1]} \phi _i$ and so by the reasoning in the proof of Theorem 7 there exists a polynomial-sized extended $\mathbf{K }_n$-Frege refutation of $MPHP_n^m$.

That the refutation size remains polynomial when replacing these extension variables with the corresponding modal formulas again follows as in the propositional case. Hence, we have a polynomial-sized $\mathbf{K }_n$-Frege proof of $MPHP_n^m$. $\square$

#### Corollary 1

There exists an exponential separation between the proof size required to refute $MPHP_n^m$ in $\mathbf{K }_n$-Frege and $\mathbf{K }_{mc}$-Res.

#### Proof

This follows immediately from Theorems 6 and 8. $\square$

Propositional separations between $\mathbf{K }_{n}$-Frege and $\mathbf{K }_{mc}$-Res follow trivially from the fact that a number of propositional formulas (including the propositional pigeonhole principle) have previously been shown to be hard for propositional resolution but easy for propositional Frege. Hence, the significance of the above result is that $\mathbf{K }_{mc}$-Res requires an exponential number of modal resolution steps to refute $MPHP_n^m$, whereas there exists a polynomially sized $\mathbf{K }_n$-Frege refutation of $MPHP_n^m$. Clearly, any polynomial-sized $\mathbf{K }_n$-Frege refutation may contain at most a polynomial number of modal proof steps (i.e. applications of $K_a$ or $\hbox {NEC}_a$) and so the separation in Corollary 1 is a truly modal one.

### Game Theoretic Lower Bound Technique vs Existing Lower Bound Techniques

In [40], Hrubeš adapted a propositional lower bound proving technique to obtain lower bounds for $\mathbf{K }_n$-Frege. This lower bound proving technique works by allowing hardness results from circuit complexity to be applied to proof complexity and is similar to well-known propositional lower bound proving technique called feasible interpolation [56].

The statement “if a graph of size *n* has a clique of size $k+1$ then it is not *k*-colourable” can be formulated as a propositional formula:

$$\begin{aligned} Clique_n^k({\bar{p}},{\bar{r}})\rightarrow (\lnot Colour^k_n({\bar{p}},{\bar{s}})), \end{aligned}$$

where:

$$\begin{aligned}&Clique_n^k({\bar{p}},{\bar{r}})=\bigwedge _j\bigvee _i r_{i,j}\wedge \bigwedge _{i}\bigwedge _{j_1\ne j_2}(\lnot r_{i j_1}\vee \lnot r_{i j_2})\bigwedge _{i_1\ne i_2, j_1 j_2}(r_{i_1 j_1}\wedge r_{i_2 j_2}\rightarrow p_{i_1 i_2}), \\&\quad \text {and }\qquad Colour^k_n({\bar{p}},{\bar{s}})=\bigwedge _i\bigvee _j s_{ij}\wedge \bigwedge _{i_1\ne i_2,i_2,j}(p_{i_1 i_2}\rightarrow (\lnot s_{i_1 j}\vee \lnot s_{i_2 j})). \end{aligned}$$

In [39, 40], a lower bound is obtained for a modal version of this formula:

$$\begin{aligned} Clique_n^k(\Box {\bar{p}},{\bar{r}})\rightarrow \Box (\lnot Colour^k_n({\bar{p}},{\bar{s}})), \end{aligned}$$

where $Clique_n^k(\Box {\bar{p}},{\bar{r}})$ denotes the formula obtained by replacing each $p_{i_1i_2}$ in $Clique_n^k({\bar{p}},{\bar{r}})$ with $\Box p_i$. Notably, the exponential lower bound obtained is on the number of *K* axioms needed to prove $Clique_n^k(\Box {\bar{p}},{\bar{r}})$. Hence, it is a modal lower bound.

As $\mathbf{K }_{n}$-Frege *p*-simulates $\mathbf{K }_{mc}$-Res, this modal clique-colour formula must also give an exponential lower bound for $\mathbf{K }_{mc}$-Res. However, as the negation of the modal clique-colour formula is of the form:

$$\begin{aligned} Clique_n^k(\Box {\bar{p}},{\bar{r}})\wedge \Diamond Colour^k_n({\bar{p}},{\bar{s}}), \end{aligned}$$

it is not hard to see that the corresponding set of $\hbox {SNF}_{{mc}}$ clauses must contain only one negative modal clause. As our game theoretic lower bound technique counts the number of distinct negative modal clauses needed to refute a formula, it clearly cannot be used to show that the modal clique-colour formula gives a lower bound for $\mathbf{K }_{mc}$-Res.

In [18], it was shown that a propositional Prover–Delayer game characterises the proof size of tree-like propositional resolution. That our game cannot be used to prove the hardness of the modal clique-colour formula illustrates that it is not a characterisation of the modal proof size of $\mathbf{K }_{mc}$-Res. It is unsurprising as our game fails to provide such a characterisation as it counts only the number of distinct negative modal clauses needed to refute a formula, not the total number of modal resolution steps required.

## Conclusion and Future Work

In this paper, we have initiated the proof complexity of modal resolution systems by (i) showing the robustness of existing [48, 50] and new modal resolution systems by establishing their equivalence through simulations; (ii) devising the first lower bound technique for these systems; (iii) illustrating the technique by a new class of formulas; and (iv) comparing resolution to the stronger modal Frege systems of [40].

We believe that these findings are just the beginning of a more comprehensive understanding of proof complexity of modal resolution. Our new Prover–Delayer game presents the first lower bound technique for modal resolution. This prompts the question whether propositional lower bound techniques such as the size-width technique [7] or feasible interpolation [43] can be lifted to modal resolution. Initial research into this direction is reported in [61].

Further, it would be interesting to obtain more modal formulas with a combinatorial structure that lends itself to a proof complexity analysis. Here we believe that our modal adaptation of the pigeonhole formulas can be used for further principles such as the clique formulas from [3, 19, 20]. Most of the benchmark formulas from [4], however, do not appear to be suited for a proof complexity analysis.

On the practical side, we hope that the research line initiated here will also contribute towards better modal theorem provers, through tight calculi (such as $\mathbf{K }_{mc}$-Res), new benchmark formulas, and their proof complexity analysis.

We mention that besides the modal resolution systems studied here, there are further modal resolution systems. In particular, the first such systems have been developed by Enjalbert and del Cerro [35]. We have concentrated here on the modal resolution systems of [48, 50] because they are technically simpler (though still quite complex), more recent, and form the basis for modal solving [51]. A partial proof complexity comparison of the system of [35] to the modal systems studied here is performed in [61], where it is shown that the system of [35] simulates the systems studied here. The reverse simulation is open, but conjectured to hold [61]. If true, this would give additional justification to focus on the modal systems studied in this paper. We also mention that there are further modal resolution systems, introduced in [1, 2]. To compare these two systems of [48, 50] is left for future work.

A further direction for future research is to explore whether our lower bound method via Prover–Delayer games is applicable to further non-classical logics. At this point, it is known to work for propositional resolution [18, 19, 57], QBF [17], and resolution for the modal logic $\mathbf{K }_n$ (shown here). There are further logics, e.g. coalition logic [53] and preferential logic [52], for which resolution systems exist and it would be interesting to explore whether similar techniques work there. In general, it appears that proof complexity of non-classical logics is at a quite early stage (cf. [23] for a survey), and a number of the existing proof-size lower bounds for some logics, such as for default logic [24, 34], autoepistemic logic [9], and circumscription [13], are somewhat ad hoc without using general techniques. It would be interesting to explore existing propositional proof complexity techniques [44] more widely in the context of non-classical logics. One such technique is feasible interpolation [43], which also (in a restricted version) applies to modal Frege [40] and modal resolution [61]. From QBF proof complexity, we know that attempting to lift propositional techniques to different logics can be challenging.^10^ We hope that our work here will trigger more research in this direction.

## Footnotes

1 Cf. also [21] for a similar discussion of genuine vs lifted bounds in QBF and on how to formalise the notion of “genuine” bounds

2 We say a propositional formula is valid (satisfiable) iff it evaluates to true under every (some) assignment to its variables.

3 A formula over the set of operators $\{\Box _a,\Diamond _a,\lnot ,\wedge ,\vee \}$ is in NFF if only propositional variables are allowed to be within the scope of $\lnot$.

4 If IRES1 or IRES2 was used to infer some $A_j\in P$ then $A_j$ would be a start clause. MRES and GEN2 can only be applied to modal clauses. GEN1 and GEN3 both require every variable in a literal clause to be resolved on simultaneously and so if either was used to infer some $A_j$ then $y_1\not \in A_j$.

5 That is, the sequence of clauses where each clause is labelled by the unified modal context of the corresponding SNF clause.

6 So for example given a model *M* we say a world *w* is $(a,(x',x))\in {\mathscr {A}}\times {\mathscr {L}}_{{\mathscr {C}}-}$-reachable from a world *u* if $(x'\rightarrow \Diamond _a x)\in {\mathscr {C}}$, the valuation $V(u)(x')=V(w)(x)=1$ and $(u,w)\in R_{a}$.

7 If *C* is a positive modal clause then $C=(e:l\rightarrow \Box _a l')$ and so *M* must contain some world *w* which is *e*-accessible from $w_{\varepsilon }$ and for which $V(w)(l)=1$, and some world $w'$ that is *a*-accessible from *w* and for which $V(w')(l')=0$. Similarly if *C* is a literal clause then $C=(e:l_1\vee \dots \vee l_z)$ and so *M* must contain a world *w* which is *e*-accessible from $w_{\varepsilon }$ and for which $V(w)(l_1)=\dots =V(w)(l_z)=0$.

8 That is, if we have a set of clauses ${\mathscr {C}}'$ to which we apply some rule of $\mathbf{K }_{mc}$-Res to obtain a clause *C*, then $(M,w)\models C$ iff $(M,w)\models {\mathscr {C}}'$.

9 That, is if $w\in W$ is labelled by some $e\in \bar{{\mathscr {E}}}_{{\mathscr {C}}}^*$ s.t. $|e|>1$ and the last symbol in *e* is of the form $(a,x)\in {\mathscr {A}}\times {\mathscr {X}}_{{\mathscr {C}}-}$ or $(a,(x',x))\in {\mathscr {A}}\times {\mathscr {L}}_{{\mathscr {C}}-}$ then $V(w)(x)=1$.

10 Lifting feasible interpolation works [15], while a straightforward lifting of the size-width relation fails [16] (cf. however also [11]).
